# Navigating the Adipocyte Precursor Niche: Cell-Cell Interactions, Regulatory Mechanisms and Implications for Adipose Tissue Homeostasis

**DOI:** 10.33696/signaling.5.114

**Published:** 2024

**Authors:** Devesh Kesharwani, Aaron C. Brown

**Affiliations:** 1 Center for Molecular Medicine, MaineHealth Institute for Research, 81 Research Drive, Scarborough, ME 04074, USA; 2 School of Biomedical Sciences and Engineering, The University of Maine, Orono, Maine 04469, USA; 3 Tufts University School of Medicine, 145 Harrison Ave, Boston, MA 02111, USA

**Keywords:** Adipogenesis, Adipose-derived stem cells, ASCs, Adipogenic precursors, Metabolic syndrome, Diabetes, Obesity, Brown adipose

## Abstract

Support for stem cell self-renewal and differentiation hinges upon the intricate microenvironment termed the stem cell ‘niche’. Within the adipose tissue stem cell niche, diverse cell types, such as endothelial cells, immune cells, mural cells, and adipocytes, intricately regulate the function of adipocyte precursors. These interactions, whether direct or indirect, play a pivotal role in governing the balance between self-renewal and differentiation of adipocyte precursors into adipocytes. The mechanisms orchestrating the maintenance and coordination of this niche are still in the early stages of comprehension, despite their crucial role in regulating adipose tissue homeostasis. The complexity of understanding adipocyte precursor renewal and differentiation is amplified due to the challenges posed by the absence of suitable surface receptors for identification, limitations in creating optimal *ex vivo* culture conditions for expansion and constraints in conducting *in vivo* studies. This review delves into the current landscape of knowledge surrounding adipocyte precursors within the adipose stem cell niche. We will review the identification of adipocyte precursors, the cell-cell interactions they engage in, the factors influencing their renewal and commitment toward adipocytes and the transformations they undergo during instances of obesity.

## Introduction

Understanding the regulation of adipose tissue holds significant clinical importance, given the association between obesity and an elevated risk of diabetes, stroke, heart disease, and cancer, leading to increased healthcare expenses and reduced life expectancy [1]. According to the Centers for Disease Control and Prevention National Diabetes Statistics Report, among US adults aged 18 years or older with diagnosed diabetes, 89.8% were overweight or obese, making this the largest risk factor for developing diabetes [2].

The global surge in obesity is at least in part attributed to the consumption of high-caloric food combined with a sedentary lifestyle [3]. Traditional approaches like diet and exercise often fall short of achieving long-term weight loss due to biological adaptations in chronically obese individuals [4]. Consequently, there is a pressing need to develop innovative therapeutic strategies to address the public health risks associated with obesity.

The onset of weight gain and obesity is driven by prolonged periods of food excess, creating a positive energy balance and excessive lipid storage in adipose tissue. This process results in inflammation, cellular stress, insulin resistance, and the potential development of diabetes [5]. Obesity triggers complex changes in adipose tissue, leading to hyperplasia and hypertrophy of adipocytes, with lipid accumulation in visceral depots due to reduced adipogenic capacity [6]. A hallmark of obesity-related adipose tissue is persistent low-grade inflammation, characterized by elevated levels of immune cells such as T cells, B cells, macrophages, neutrophils, and mast cells [7]. In lean adipose tissue, M2 macrophages dominate, conferring anti-inflammatory effects, while in obesity, M1 macrophages prevail, releasing pro-inflammatory cytokines that induce insulin resistance [8]. Depletion of adipose tissue macrophages improves insulin sensitivity, with visceral adipose tissue showing a higher recruitment of pro-inflammatory macrophages compared to subcutaneous tissue [9–11]. Additionally, obesity leads to a decrease in regulatory CD4 helper T cells and an increase in CD8 T cells in visceral adipose tissue [12–14]. Eosinophils play a role in adipocyte browning, and their loss during obesity exacerbates diet-induced weight gain [15]. Obesity also reduces adipose tissue capillarization, potentially worsening insulin resistance and adipocyte dysfunction [6]. Increasing VEGF-mediated angiogenesis in adipose tissue can mitigate metabolic consequences of obesity, such as insulin resistance and hepatic steatosis [16,17]. In addition, adipose tissue inflammation is fueled by heightened secretion of TNF-α and free fatty acids from enlarged adipocytes, which impairs insulin sensitivity and triggers adipocyte lipolysis [18]. Moreover, TNF-α compounds insulin resistance by suppressing key genes involved in adipocyte development and insulin signaling [19]. These disruptions in adipose tissue function not only contribute to the prevalence of non-alcoholic fatty liver disease in obese, insulin-resistant, and diabetic individuals but also impact insulin secretion by inhibiting β-cell potassium channels and altering ATP production [18]. Meanwhile, the adipokine profile of visceral white adipose tissue (WAT) serves as a critical determinant of cardiovascular disease (CVD) risk, with adiponectin playing a protective role through cholesterol reduction, inflammation suppression, and AMPK activation [20–22]. Conversely, lower plasma adiponectin levels in CVD patients, along with the presence of resistin and FGF21, signify elevated risk factors associated with obesity, type 2 diabetes, and atherosclerosis development, driven by increased TNF-α levels in serum and adipose tissue [23–27].

During healthy conditions, adipose tissue serves multiple functions, including structural support to organs, protection from cold, and crucially, regulating energy balance and metabolic homeostasis [28]. Both humans and rodents possess white adipose tissue (WAT) responsible for energy storage, with its accumulation correlating with metabolic syndrome in overweight individuals. Conversely, brown adipose tissue (BAT) converts stored lipids into heat and exhibits a positive correlation with a reduced risk of metabolic syndrome, making it a promising therapeutic target [1]. Apart from the conventional BAT depot situated in the interscapular region, functionally analogous “beige” adipose tissue can be induced within adult subcutaneous white adipose tissue (WAT) in response to cold-induced norepinephrine secretion via the sympathetic nervous system. The generation of beige adipose tissue within subcutaneous WAT signifies a dynamic adaptation, revealing the plasticity of adipose tissue and its ability to respond to environmental cues by adopting characteristics similar to those of thermogenic BAT [29]. Gaining insight into the biological mechanisms governing the development and maintenance of white, brown, and beige adipose tissues is crucial for developing targeted interventions and therapeutic strategies to prevent and treat metabolic disorders.

Within a tissue, a stem cell niche establishes a specialized microenvironment where direct cell-cell interactions and molecular signals sustain stem cells in an undifferentiated state or facilitate their differentiation. In particular, the adipose stem cell niche comprises various cell types, including adipocytes, multipotent adipose-derived stem cells (ASCs or ADSCs), committed adipocyte progenitors (APs), endothelial cells, fibroblasts, immune cells, and vascular smooth muscle cells. These cells communicate through direct interactions or paracrine signaling via adipokine secretion [30]. This review provides an overview of the adipose stem cell niche, with a focus on characterizing ASCs and APs, exploring their interactions with other cell types in the niche, examining their molecular regulation, and investigating the changes they undergo in response to increased adiposity, which may contribute to the development of obesity-related disorders.

## Characterization and Significance of the Adipose Tissue Stromal Vascular Fraction

The stromal vascular fraction (SVF) refers to a heterogeneous mixture of cells that can be isolated from adipose tissue through enzymatic digestion. This fraction excludes mature adipocytes and consists of a variety of cell types, including ASCs, APs, fibroblasts, immune cells, endothelial cells, epithelial cells, and other cells associated with the circulatory and nervous systems [31]. The SVF is particularly rich in stromal and vascular components, and its diverse cell population makes it a valuable source for regenerative and therapeutic applications in medical research and clinical settings [32]. Stem cells and progenitor cells are frequently used interchangeably, yet their definitions are contentious and continue to evolve [33]. Stem cells are characterized by distinct features such as pluripotency and the capacity for unlimited replication. Conversely, progenitor cells, while still possessing replicative abilities, are more limited in replication and are further along the differentiation path as they have committed to a specific lineage. The absence of appropriate culture conditions for assessing replicative capacity and the scarcity of distinctive surface markers have posed challenges in differentiating between stem and progenitor cells originating from the adipose tissue niche. In the context of this review, the term ASCs will specifically denote the subset of mesenchymal stem cells (MSCs) residing in adipose tissue, possessing multilineage potential to differentiate into adipocytes, osteoblasts, chondrocytes, and other lineages, which constitute less than 0.1% of all SVF cells [34,35]. On the other hand, APs, also known as preadipocytes, will refer to cells committed to the adipocyte lineage, typically constituting 15%−35% of the SVF [34–37]. It’s crucial to note that APs frequently exhibit a similar cell surface phenotype to ASCs (unless specified otherwise), although they might display markers indicating commitment to the adipocyte lineage, such as expression of PPARγ [35]. Finally, within this review, the term “adipocyte precursors” will broadly encompass any cell within the niche possessing the potential to become adipogenic, encompassing ASCs, adipocyte progenitors, and potentially other cell varieties.

## Adipose Tissue Dynamics, Differentiation, and Metabolic Regulation

Adipose tissue exhibits a distinctive capacity to expand and contract in response to various physiological conditions, including instances of overeating, dietary changes, and physical exercise. This dynamic nature underscores its crucial role in energy storage and metabolic regulation within the body. Additionally, the adaptability of adipose tissue plays a key role in maintaining overall homeostasis and responding to the fluctuating energy demands imposed by different lifestyle and dietary factors. In general, the expansion of subcutaneous WAT is linked to a lower susceptibility to cardiometabolic syndrome during obesity, highlighting its potential protective role. Conversely, the expansion of visceral WAT is frequently correlated with insulin resistance and an increased risk of developing diabetes [38,39]. This distinction underscores the importance of considering not only the overall adipose tissue mass but also the specific depots, as their responses may have distinct implications for metabolic health. The enlargement of adipose tissue mass can result from hyperplasia, involving an augmentation in the number of adipocyte cells, which is generally considered more metabolically advantageous than hypertrophy, where adipocyte cell size increases due to heightened lipid storage [40]. In adults, the quantity of adipocytes remains relatively consistent in both lean and obese individuals, with approximately 10% of mature adipocytes being replenished annually through the differentiation of adipocyte precursors [41,42].

The initial stage of adipocyte differentiation is initiated when ASCs respond to external signals, prompting them to transform into APs with a specific commitment to the adipocyte lineage. Subsequently, in the second phase, the terminal differentiation of committed APs into fully mature adipocytes, characterized by cell cycle arrest, involves a coordinated series of transcriptional events primarily governed by CCAAT-enhancer-binding proteins (C/EBPs), sterol regulatory element binding protein 1c (SREBP1c) and peroxisome proliferator-activated receptor gamma (PPARγ) [43–46]. PPARγ serves as the principal regulator of adipogenesis, playing a pivotal role in orchestrating the process. Ablation of PPARγ has been shown to hinder adipogenesis and prevents the development of adipose tissue in mice [47,48]. C/EBPs and SREBPc1 induce the expression of PPARγ, which then directly interacts with retinoid X receptor (RXR). This interaction enables the binding of the PPARγ-RXR complex to responsive regulatory elements, leading to the activation of genes involved in adipogenesis, lipid metabolism, inflammation, and the maintenance of metabolic homeostasis [46,49,50]. Upon formation, mature adipocytes acquire the molecular machinery required for lipid and glucose transport, insulin responsiveness, and the secretion of paracrine factors, collectively contributing to the regulation of systemic metabolic homeostasis [43].

In contrast to white adipocytes, brown and beige adipocytes exhibit distinct transcription factors that govern their differentiation and function. These include the expression of PR domain zinc finger protein 16 (PRDM16), early B cell factor 2 (EBF2) and peroxisome proliferator-activated receptor gamma coactivator 1-alpha (PGC-1α) (reviewed in Ref. [44]). These transcription factors, in collaboration with PPARγ, orchestrate an elevation in the expression of uncoupling protein-1 (UCP1) and facilitate the activation of UCP1-mediated thermogenic responses in response to β-adrenergic receptor stimuli.

## Advantages of Immortalized Adipocyte Precursor Cell Lines

The notion that adipocytes could emerge from a pool of stem/progenitor cells gained prominence during the refinement of techniques for segregating adipose tissue into adipocytes and the SVF (reviewed in [31]). These investigations revealed that adherent fibroblast-like cells within the SVF exhibited competence for adipogenesis, leading to their subsequent classification as preadipocytes [51–53]. As the SVF can give rise to cell types beyond adipocytes, such as osteoblasts, chondrocytes, and myoblasts, these cultures likely represent the coexistence of multipotent MSCs alongside solely committed APs [31]. For a deeper understanding of adipocyte differentiation and cellular physiology, as well as to address challenges linked to the variable differentiation and premature senescence of SVF cells, numerous immortalized, clonal AP cell lines have been developed. For instance, immortalized 3T3-L1 cells, developed from mouse embryonic fibroblasts in the 1970s, are recognized as a committed AP cell line. These cells were instrumental in elucidating that the transcription factor PPARγ acts as the primary regulator of adipocyte differentiation and collaborates with C/EBPα to stimulate adipogenesis [54–57]. 3T3-L1 APs are generally acknowledged as committed to the development of mature white adipocytes and are thought to possess a limited capacity for thermogenesis. However, the extent of this thermogenic potential has been a topic of debate since the cells were first derived [58]. Cell lines like the mouse-derived HIB-1B and “Thermomouse” lines, originating from interscapular BAT, exhibit a robust thermogenic program, serving as invaluable tools to dissect mechanisms involved in brown adipose tissue thermogenesis [59,60]. The extent to which these immortalized cell lines accurately represent a specific progenitor type found within the native adipose tissue niche, when compared to primary isolated adipocyte progenitors APs from distinct adipose tissue depots, remains unclear.

## Challenges in Identifying Adipocyte Precursors through Immunophenotyping

The predominant approach for discerning adipocyte precursors from other cell types in the adipose tissue niche has been through the analysis of cell surface markers. However, identifying, and characterizing adipocyte precursors within the SVF has posed challenges for several reasons. Firstly, diverse developmental origins are known to give rise to anatomically distinct adipocytes, including white, beige, and brown subtypes. This implies that specific developmental programming may lead to distinct precursor lineages, each giving rise to a particular type of adipocyte. Additionally, during SVF culture, surface marker expression may undergo changes, complicating the establishment of a definitive marker profile [31]. Discrepancies in surface marker expression have also emerged between human and rodent studies, and numerous surface markers may lack specificity for adipocyte precursors, being expressed on non-stem cell lineages [31]. Moreover, surface marker profiles may vary or overlap between ASCs and more committed APs. These complexities contribute to the challenge of establishing a definitive adipocyte precursor cell surface marker profile. Nonetheless, several markers have proven valuable in studying these cells and enriching populations with substantial adipogenic potential.

CD34 serves as a prevalent MSC marker expressed on ASCs exhibiting high adipogenic potential. Utilizing CD34, in conjunction with depleting common endothelial (CD31) and hematopoietic (CD45) markers, proves effective in enriching ASCs from the SVF [61,62]. However, potential inconsistency in results with CD34 may arise due to its downregulation post-cell culture [31,63]. CD29 and Sca1 (specific to mice) are also recognized as common MSC markers suitable for identifying and enriching ASCs. Notably, Friedman *et al*. outlined a subset of undifferentiated ASCs marked by CD29+, CD34+, Sca-1+, CD24+, demonstrating the capability to proliferate and differentiate into adipocytes [34]. These precursors are also capable of restoring a fully functional and normal WAT depot and effectively reverse diabetic phenotypes seen in lipodystrophic mice [34]. CD24 has been employed to distinguish hierarchical populations of adipocyte precursors, revealing varying levels of commitment and potential [64]. In adipose depots displaying a hyperplastic response, CD24+ ASCs undergo rapid and transient proliferation shortly after the initiation of a high-fat diet [65]. Furthermore, CD24+ ASCs represent a more primitive multipotent stem cell population. As cells initiate the expression of adipocyte lineage-selective genes like *Pparγ* and *C/ebpα*, indicative of a more committed stage in adipogenesis, CD24 expression is lost [64,66].

## Adipocyte Precursors and Their Heterogeneity within the Adipose Tissue Microenvironment

### Adipocyte precursor positioning within the extracellular matrix of adipose tissue

**Figure 1:**
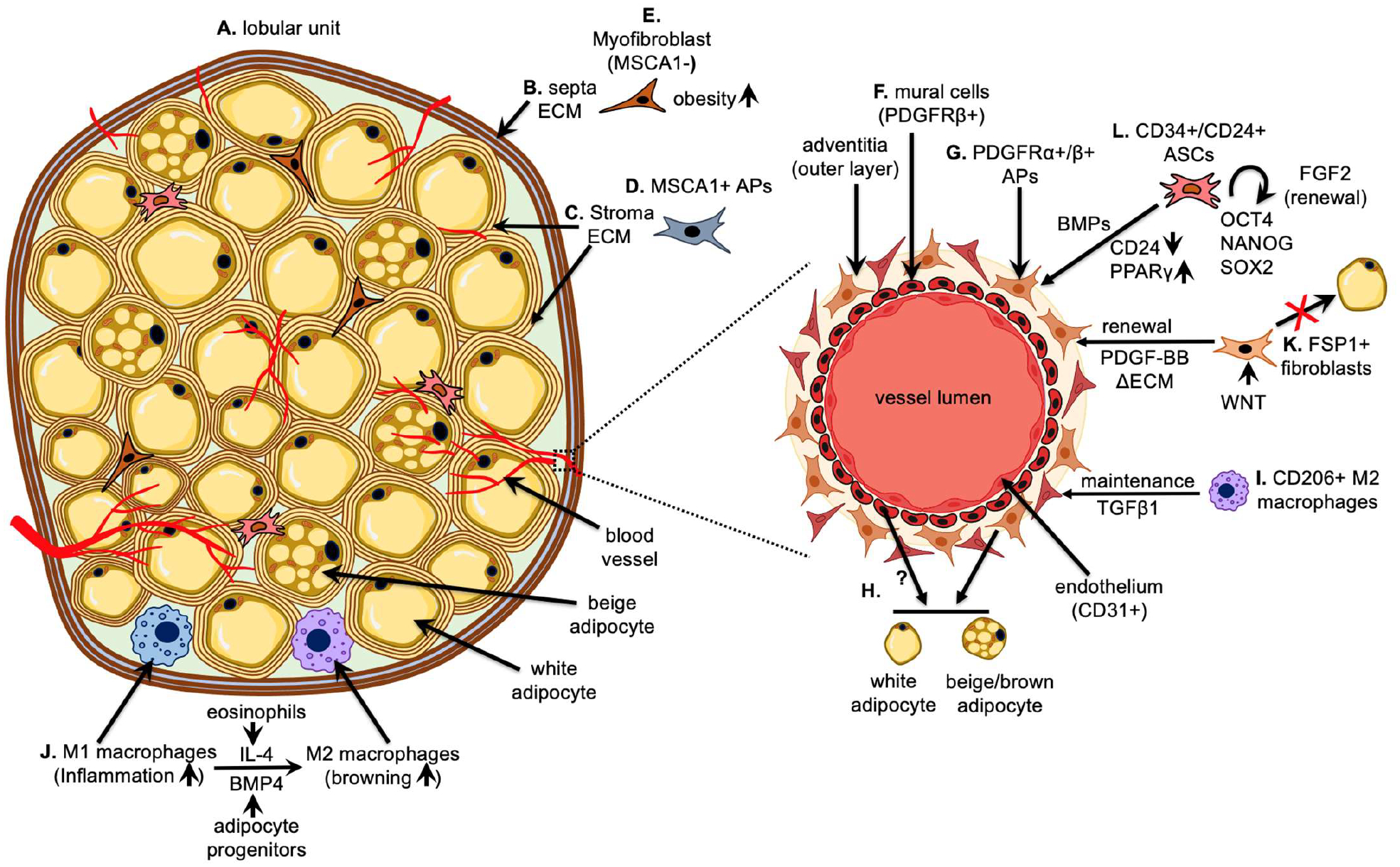
Structure and interplay within the adipose tissue microenvironment. (**A**) Adipose tissue consists of clusters of adipocytes arranged in lobules, which are separated from each other by ECM that forms the septa (**B**). In addition to the septa lining the outside of the lobule, the internal stroma also contains a distinct ECM compartment (**C**). (**D**) ECM within the stroma of the lobule contains CD34+/MSCA1+/CD271- APs that are highly adipogenic and positioned between adipocytes or adjacent to the vasculature. (**E**) ECM within the septa contains CD34+/MSCA1-/CD271+ myofibroblasts that may aid in formation of the fibrous septa and contribute to fibrosis during obesity. (**F**) PDGFRβ+ mural cells located in the vessel walls may harbor adipogenic activity, but recent data contradicts this and suggests PDGFRα+/β+ APs (**G**) located within the vessel wall adventitia contribute to the majority of adipocytes found in white, beige and brown adipocytes (**H**). (**I**) CD206+ M2 macrophages play a role in maintenance of APs through secretion of anti-adipogenic TGFβ1, which prevents exhaustion of the AP pool by limiting excessive proliferation and premature senescence. (**J**) IL-4 and BMP4 represent two potential factors that may decrease the proportion of M1 macrophages in adipose tissue in favor of anti-inflammatory M2 macrophages, which play a role in adipose tissue browning, protection from high-fat diet-induced obesity and improved metabolism. (**K**) FSP1+ fibroblasts found in the loose connective tissue outside the vessel wall rely on WNT signaling for proper function and lack adipogenic potential (red X). They contribute to the renewal and differentiation potential of APs through the secretion of PDGF-BB and remodeling of the ECM. (**L**) CD34+/CD24+ ASCs express markers of pluripotency (OCT4, NANOG and SOX2) and undergo FGF2 mediated self-renewal. BMP ligands can induce multipotent ASCs to form committed APs (CD24-) that express adipocyte lineage-selective genes such as PPARγ. Figure modified from [216].

In the adult, adipose tissue comprises distinct lobules characterized by clusters of adipocytes separated by extracellular matrix (ECM), forming defined partitions known as septa [67] (Figures 1A and 1B). Both mathematical modeling and experimental findings propose that these lobules spontaneously emerge through mechanical interactions between adipocytes and fibers within the ECM [68]. Recent evidence indicates that adipose tissue lobules consist of two structurally distinct ECM compartments: the septa, lining the exterior of the lobule, and the internal stroma ECM (Figure 1C) [69]. These compartments house unique subsets of CD34+ precursor cells, with a highly adipogenic committed MSCA1+/CD271− progenitor subset enriched in the stroma, and an MSCA1−/CD271^hi^ subset present in the septa, marking myofibroblast precursors potentially contributing to fibrous septa formation (Figures 1D and 1E) [69]. In the stroma, APs are discernible from pericytes and are positioned not within capillary walls but between adipocytes or in proximity to the vasculature [69]. Notably, in humans, inherent differences exist within these progenitor subsets concerning their adipogenic and myofibroblastic capacities in stroma and septa niches. This includes a higher myofibroblastic capacity in visceral WAT compared to subcutaneous WAT during obesity, potentially contributing to fibrosis [69].

### Adipocyte precursors within the mural cell compartment

Early morphological investigations have indicated the close association of adipocyte development with the vasculature, prompting the proposal that pericytes, a subset of mural cells known for modulating endothelial cell functions, vessel contractility, and possessing multipotent MSC characteristics, may also serve as a precursor population for adipocytes (Figure 1F) [67,70–72]. In their quest to identify committed APs within the adipose tissue niche, Tang *et al*. postulated that these cells would exhibit the expression of PPARγ, a pivotal regulator in the formation of adipocytes [37,73]. Using a *Pparγ* reporter strain, they demonstrated that *Pparγ*+ cells are present in PECAM+ (CD31) blood vessel walls of WAT, exhibiting high adipogenic potential and expressing mural cell markers, including α-SMA, PDGFRβ, and NG2 [37]. Most of the committed *Pparγ+* APs co-expressed Sca1 and CD34, but lacked expression of CD105 (MSCs), CD45 (immune cells), TER-119 (erythrocytes) or Mac-1 (monocytes). Notably, APs expressing Pparγ from the mural cell compartment were absent in the vessel walls of other tissues (such as skeletal and cardiac muscle, kidney, retina, pancreas, spleen and lung). Furthermore, the mural cells within these additional tissues did not demonstrate a notable capacity for high adipogenic potential [37]. Moreover, lineage tracing experiments provided evidence that adipose tissue can originate from mural cells expressing *Pdgfrβ*. When transplanted into nude mice, these *Pdgfrβ*+ mural cells exhibited substantial adipogenic potential [37]. In a subsequent investigation employing a doxycycline-inducible tracking system for mural cell lineage based on *Pdgfrβ* expression, it was demonstrated that these perivascular APs also play a role in promoting adipocyte hyperplasia in visceral WAT during high-fat diet feeding [74]. In summary, these findings indicate the presence of a distinct population of APs situated within the mural cell compartment of adipose tissue.

Recent research has utilized Cre drivers specific to vascular smooth muscle cells (*Myh11*, *α-Sma*) and mural cells (*Pdgfrβ*) to reveal that certain subgroups of beige adipocytes can also be traced back to the mural cell compartment [74–76]. In our laboratory, research has been directed towards the generation of beige adipocytes from induced pluripotent stem cells (iPSCs) [77]. These adipocytes originate from the splanchnic mesoderm, an embryonic tissue recognized for its capacity to generate mural and vascular smooth muscle cells [78]. Our results showed that MSCs originating from the splanchnic mesoderm exhibit positivity for *α*-SMA, PDGFRβ and NG2. These MSCs give rise to UCP1+ adipocytes that showcase the distinctive gene expression profile associated with beige adipocytes (CITED1+, CD137+, TMEM26+). Notably, this gene expression signature differs from that of classical brown adipocytes, which typically express ZIC1 and a myogenic skeletal muscle signature [77,78]. Remarkably, beige adipocyte precursors originating from mural cells (α-SMA+) in mice display a senescence-like phenotype by the age of 6 months, hindering beige adipocyte formation during cold exposure [79]. Corresponding outcomes in this study were observed in beige precursors derived from elderly human patients. Intervening pharmacologically in the senescence-like process proved effective in revitalizing dysfunctional aged beige precursors, enabling them to resume beige adipogenesis. In mice, this intervention resulted in a simultaneous reduction in blood glucose levels. This underscores the significance of identifying specific subgroups of adipocyte precursors for targeted interventions aimed at enhancing metabolic health.

### Adipocyte precursors derived from fibroblastic cells

Further lineage tracing investigations have revealed the presence of additional APs that exist beyond the confines of the mural cell compartment. PDGFRα serves as a broad fibroblast marker found on cells identified as progenitors across diverse mesenchymal tissues [80]. In WAT, progenitors marked by PDGFRα constitute a subset within the adipocyte precursor compartment characterized by CD34 and Sca-1, displaying bipotential capabilities [36]. These cells undergo proliferation and have the capacity to generate white adipocytes under normal conditions or during high-fat feeding. Alternatively, upon pharmacological stimulation of the β3-adrenergic receptor (ADRB3), they can produce beige adipocytes [36]. It has been observed that PDGFRα+ APs are situated in proximity to the vasculature, featuring extensive dendritic processes that establish contact with multiple cells in the tissue environment [36]. In this study, PDGFRα+ APs in WAT were demonstrated to lack expression of PPARγ, α-SMA and PDGFRβ, indicating their location outside the mural cell compartment [36]. Notably, in a subsequent investigation, the researchers illustrated that APs marked by PDGFRα+ in subcutaneous WAT could be prompted to undergo differentiation into beige adipocytes through acute cold exposure (1 week) [81]. Conversely, it has been demonstrated that a duration of two weeks of cold exposure is required to facilitate the development of beige adipocytes from mural APs marked by PDGFRβ [74,75]. Thus, adipose precursors identified by PDGFRα+ in WAT may be functionally differentiated from those in the mural cell compartment by their capacity to promptly react to metabolic stress. Consequently, beige adipogenesis might hinge on various adipocyte precursor populations, each with their own specific timing and activation events [64].

Newly available information is elucidating the distinctions between mural (PDGFRβ+) and fibroblastic (PDGFRα+) sources of adipogenic precursors, along with their respective roles in adipose tissue development under conditions such as high-fat diet, browning, and regular metabolic homeostasis [82]. By employing three distinct Cre drivers—Tie2-Cre for hematopoietic and endothelial cells, Tbx18Cre-ERT2 for mural cells (pericytes and vascular smooth muscle cells), and Pdgfrα-MerCreMer for adipose tissue fibroblasts—it was demonstrated that only adipose tissue fibroblasts located within the blood vessel adventitia and adipose tissue capsule regions were notably involved in *de novo* adipogenesis among the various cell types within the vascular wall [82]. Significantly, these findings illustrated that PDGFRβ not only identifies mural cells but also marks adventitial PDGFRα+ APs, potentially providing insights into the reasons behind previous studies indicating a potential involvement of mural cells (Figure 1G) [82]. Additional investigations corroborate this inference, as single-cell RNA sequencing has revealed that APs express both PDGFRα and PDGFRβ [83]. The findings from our research using multipotent, human iPSC-derived mural cells also indicated that as these cells transitioned into committed APs, they gained PDGFRα expression concurrent with heightened PPARγ expression [77]. Nevertheless, it is yet to be elucidated whether PDGFRβ+ mural cells have the capacity to generate double-positive PDGFRα/PDGFRβ APs in the course of *in vivo* adipocyte development. Additionally, there is a need to investigate potential variations in the expression of these markers between mice and humans during *de novo* adipogenesis. Utilizing single-cell RNA sequencing analysis to identify beige adipocyte precursors, Oguri *et al*. discovered that CD81 serves as a marker for a novel subset of PDGFRα+ stromal cells expressing a smooth muscle-like signature, including α-SMA, *Sm22*, *Myl9*, and *Myh11* [84]. This subset of APs is characterized by high proliferative activity and gives rise to beige adipocytes. CD81 was also identified as a functional regulator of beige adipocyte precursor proliferation through irisin-mediated FAK signaling. In murine models, CD81 was demonstrated to be essential for *de novo* beige fat biogenesis, and its deletion resulted in diet-induced obesity, glucose intolerance, and adipose tissue dysfunction. Additionally, in humans, a decreased count of CD81^+^ adipocyte precursors in subcutaneous WAT was shown to be indicative of metabolic risk, including elevated fasting blood glucose levels, diastolic blood pressure, visceral fat mass, and visceral adiposity. Overall, the exploration of cell surface marker profiles linked to different subsets of adipocyte precursors is an ongoing process, evolving as newly identified populations continue to emerge.

## Adipocyte Precursor Interactions with Other Cells and Their Influence within the Adipose Tissue Niche

Adipocyte precursors are situated within the perivascular region and stromal component of adipose tissue lobules, engaging in interactions with both mature adipocytes and other cells within the precursor niche *in vivo*. Our comprehension of the impact of these interactions on the fate of adipocyte precursors and, consequently, adipose tissue homeostasis is still in its nascent stages. In response to various homeostatic and external signals such as weight gain, hypoxia, cold, exercise, and nutrition, mature adipocytes undergo activation and release adipokines that have the potential to influence both energy intake and expenditure [85] and adipocyte precursor differentiation [86]. Through experiments with cultured adipocytes or adipose tissue explants, it has been revealed that secreted factors from mature adipocytes can exert either positive or negative regulation on adipocyte differentiation [86]. Furthermore, in these studies, the combined secretome of adipose tissue collectively led to the overall inhibition of AP differentiation. It is important to note that, apart from mature adipocytes, other cells within the adipose tissue niche undoubtedly contribute to these processes.

The primary focus of research on cell-cell interactions promoting the maintenance, proliferation, or commitment of adipocyte precursors toward differentiation into mature adipocytes has predominantly centered around cells within isolated SVF. Nonetheless, the mechanism by which the SVF and the adipose tissue niche collectively govern the size and fate determination of the adipocyte precursor pool, thereby influencing adipocyte size, number, and overall adipose homeostasis, remains unclear [87,88]. Several constraints in performing this research involve the sharing of markers among various cell populations within the niche, resulting in challenges in distinguishing distinct cell types. Furthermore, the constitution of the separated SVF undergoes substantial alterations during tissue culture expansion, even during the initial stages of passage [89]. The primary subsets of nucleated cells residing within the SVF encompass hematopoietic, endothelial, and stromal cells; yet, notable heterogeneity exists among these populations. This diversity is notably influenced by distinct factors, including the specific anatomical localization of adipose tissue, divergent methodologies in tissue processing and culture and the unique health or metabolic constitution of the individual [89,90]. For instance, when examining adipocyte precursors isolated from the subcutaneous WAT of individuals with obesity, there emerges an upregulation in the expression of inflammatory genes. This heightened expression correlates with a diminished stemness capacity and an elevated inclination toward committing to adipocyte differentiation [91]. Additionally, adipocyte precursors sourced from the subcutaneous WAT of obese patients display a compromised ability to expand and generate beige adipocytes upon induction in cell culture [77,92]. These observations highlight the impact of the adipose tissue microenvironment on cellular behavior, particularly in contexts of obesity and altered metabolic states.

### Reciprocal interplay between adipocyte precursor and endothelial cell specialization

Due to the proximity of adipocyte precursors to endothelial cells and the integral association between adipose tissue development and angiogenesis, these cell types may undergo regulation via direct cell-cell interactions or through paracrine signaling from endothelial cells [93]. This relationship is further exemplified by the proposition that angiogenesis actively recruits adipocyte precursors and induces their differentiation [94]. Moreover, *in vitro* co-culture experiments involving endothelial cells and mature adipocytes have demonstrated a facilitative effect on the development of immature preadipocytes, concurrent with amplified growth of mature adipocytes, which appears to be an indirect consequence of the adhesion between endothelial cells and mature adipocytes [95]. The interaction between adipocyte precursors and endothelial cells seems to be bidirectional. Adipocyte precursors play a crucial role in promoting endothelial cell proliferation and differentiation through the secretion of proangiogenic factors, thereby facilitating the formation of blood vessels [89,96]. This interaction is further underscored by the discovery that multipotent CD34+ ASCs, isolated from the SVF and characterized by co-expression of mesenchymal (CD90), pericyte (PDGFRβ), and smooth muscle (α-SMA) markers, contribute to the stabilization of the vasculature. These ASCs exhibit a structural and functional association with endothelial cells, leading to enhanced stability of endothelial networks, including improved cord formation [97]. Overall, these findings suggest a collaborative and reciprocal relationship between adipocyte differentiation and angiogenesis.

### Maintenance and commitment of adipocyte precursors via immunomodulation

Immune cells are integral to the functioning of adipose tissue in both health and disease. In a healthy state, immune mechanisms are essential for maintaining tissue homeostasis through a delicate balance of cell types and signaling pathways, regulating inflammation, supporting tissue repair, and preserving metabolic health [98]. The enlargement of adipose tissue during the progression of obesity includes the accumulation of immune cells associated with chronic inflammation and disrupted metabolism, which progressively extends systemically, contributing to insulin resistance and metabolic disorders [5]. The secretion of cytokines and growth factors by immune cells may also create a conducive environment for the neovascularization of adipose tissue experiencing ischemia [89]. Despite these correlations, the precise function of immune cells within the adipocyte precursor niche during healthy (lean) and diseased (obese) states still requires further elucidation [99]. Similar to ASCs, immune cells in the SVF are CD34 positive, but can be discerned from adipocyte precursors by their inability to adhere to culture dishes, resulting in their exclusion during cell culture passage [89]. Moreover, distinguishing between immune cells and endothelial cells can be accomplished by observing the absence of the endothelial cell surface marker CD31 in immune cells [90]. These distinctions enable researchers to selectively identify and analyze specific immune cell populations, thereby enhancing the precision of understanding their roles within the adipose tissue microenvironment.

**Figure 2:**
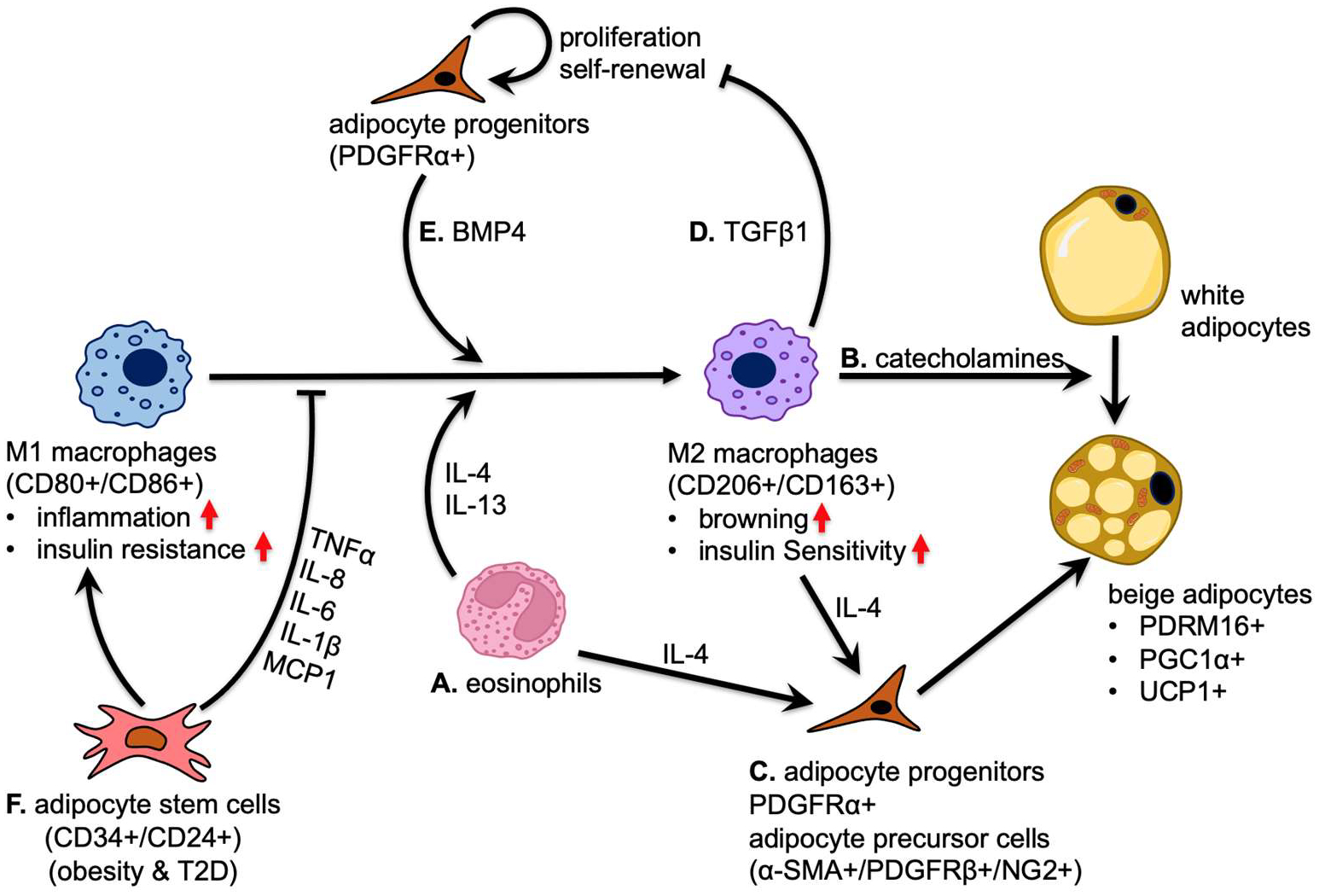
Interactions between macrophages and adipogenic precursors. (**A**) Eosinophils secrete IL-4 and IL-13 that polarize macrophages toward an M2 fate, potentially contributing to browning and the formation of beige adipocytes through catecholamine secretion (**B**). (**C**) Secretion of IL-4 from eosinophils and potentially M2 macrophages may directly impact adipogenic precursors to enhance their capacity to undergo beige adipogenesis. (**D**) TGFβ1 expressed by CD206+ M2 macrophages hinders the proliferation of PDGFRα+ adipogenic precursors to prevent premature senescence and precursor cell exhaustion. (**E**) BMP4 derived from adipogenic precursors stimulates the proliferation of CD206+ M2 macrophages while inhibiting M1 macrophages, resulting in a substantial rise in M2 macrophage numbers that may lead to increased browning of WAT. (**F**) ASCs from obese and type 2 diabetic individuals exhibit up-regulation of inflammatory genes that may contribute to adipose tissue inflammation and insulin resistance by attracting and directing macrophages towards the M1 subtype.

Multiple research investigations have demonstrated that cells belonging to the monocyte lineage, such as eosinophils and macrophages, play significant regulatory roles within the adipose tissue niche. Among these, macrophages stand out as the predominant immune cell lineage in adipose tissue, contributing to various functions, including tissue repair, insulin sensitivity, fibrosis, and metabolic homeostasis [99]. These macrophages are classified as (M1) pro-inflammatory macrophages, exacerbating insulin resistance, and (M2) anti-inflammatory macrophages, enhancing insulin sensitivity [100–102]. Adipocyte precursors and macrophages engage in interactions, with reported findings indicating that M1 pro-inflammatory macrophages regulate the expression of angiogenic genes in preadipocytes [103,104]. Furthermore, conditioned media from adipose tissue macrophages has been shown to reduce the differentiation capacity of human subcutaneous APs, as evidenced by decreased expression of key adipogenic genes PPARγ2 and C/EBPα [105]. Factors derived from pro-inflammatory macrophages, including cytokines like IL-1β and TNFα, may suppress adipogenesis by inhibiting PPARγ expression via NF-κB activation [105]. Acutely activated macrophages induce NF-κB activation in APs, possibly due to high levels of TNFα and IL-6, which may increase fibronectin and promote cell proliferation through cyclin D1 induction [105]. These findings suggest potential molecular links between macrophage-induced inflammation and altered AP differentiation. Alternative subpopulations of M2 macrophages have been associated positively with beige adipogenesis, implying distinct roles within adipose tissue biology. Cold exposure in mice triggers eosinophil activation in adipose tissue, leading to the secretion of IL-4 and IL-13 cytokines by these cells (Figures 1J and 2A) [106]. This secretion polarizes macrophages toward an M2 fate, potentially contributing to the formation of beige adipocytes through catecholamine secretion (Figure 2B) [107]. The potential of M2 macrophages to produce enough catecholamines to induce browning of white adipose tissue (WAT) has been a subject of inquiry [108]. Despite this, IL-4 secretion by immune cells may directly impact APs from subcutaneous white adipose tissue in mice, fostering beige adipogenesis (Figure 2C) [109,110]. Similarly, our laboratory has shown that treating human mural-like adipocyte precursors (α-SMA+/ PDGFRβ+/NG2+) in culture with IL-4 significantly enhances their capacity to generate beige adipocytes (Figure 2C) [77]. Additionally, recent research by Nawaz *et al*. has shown that CD206+ M2 macrophages contribute to preserving the adipogenic precursor pool by preventing exhaustion through overproliferation (Figure 1I) [111]. This involves keeping APs in a state of hibernation, preventing unnecessary cell division and potential cell senescence. Specifically, their study revealed that the presence of TGFβ1, expressed by CD206+ M2 macrophages, hinders the proliferation of PDGFRα+ adipogenic precursors (Figure 2D). This observation aligns with previous research indicating that TGFβ signaling serves as a recognized inhibitor of adipogenesis and a suppressor of subcutaneous WAT browning [112,113]. Moreover, the depletion of CD206+ M2 macrophages in mice leads to increased browning of WAT in response to cold exposure [111]. In summary, the findings from these studies illustrate that M2 macrophages have the potential to impact the adipocyte precursor niche, promoting the maintenance and commitment decisions of adipogenic precursors.

### The role of non-adipogenic fibroblasts in adipocyte precursor homeostasis

Investigations in murine models have elucidated the significance of FSP1-expressing fibroblasts as a vital cell type within the niche, crucial for the maintenance and adipogenic potential of APs (Figure 1K) [87]. These FSP+ fibroblasts express α-SMA and vimentin, are non-adipogenic, and reside in close proximity to PPARγ+ APs. WNT signaling, pivotal for adipose tissue homeostasis, operates by activating β-catenin and inhibiting AP differentiation [114,115]. Activating WNT signaling in FSP1+ fibroblasts led to a gradual reduction in adipose tissue and resistance to diet-induced obesity. This correlated with decreased expression of platelet-derived growth factor (PDGF-BB), vital for maintaining the AP pool. Restoring PDGF-BB levels increased AP percentages and their adipogenic potential. Reduced PDGF-BB signaling affected AP adipogenic capacity by altering how FSP1+ fibroblasts regulated MMP expression and remodeled the ECM in the microenvironment. Thus, FSP1+ fibroblasts play a pivotal role in maintaining adipose tissue homeostasis by creating a microenvironment that governs AP maintenance and adipogenic potential [87].

## Fibroblast Growth Factor Maintenance of the Adipocyte Precursor Pool

As ASCs exhibit potential in various regenerative applications, extended culture periods are frequently required to attain an adequate quantity for clinical use. In the cultivation of various stem cell types, FGF-2 is commonly added to the culture medium to enhance the maintenance of self-renewal capacity and plasticity across multiple passages (Figure 1L) [116,117]. Culturing adipocyte precursors for extended durations results in diminished capabilities for proliferation, self-renewal, and differentiation. This decline is linked to a reduction in FGF-2 expression by adipocyte precursors, a phenomenon that can be reversed through ongoing treatment with recombinant FGF-2 [117]. In alignment with this observation, adipocyte precursors exhibit expression of fibroblast growth factor receptor 1 (FGFR1), displaying a heightened affinity for FGF-2. Blocking this receptor leads to diminished proliferation and deactivation of kinases such as AKT, ERK, JNK, and p38 [118,119]. Supplementing adipocyte precursors with FGF-2 before exposure to an adipogenic differentiation cocktail enhances the expression of PPARγ, increasing their capacity to promote adipocyte differentiation [116]. Notably, FGF-2 produced by adipocyte precursors is exported to the cell surface without being released into the culture medium, indicating the presence of a functional autocrine loop [117]. This becomes pertinent in the context of metabolic disease, as adipocyte precursors derived from subcutaneous and visceral WAT of individuals undergoing bariatric surgery exhibit diminished FGF-2 exportation, which is associated with reduced proliferation, clonogenic potential, and unfavorable metabolic profiles [120]. It is noteworthy that the beneficial effects of FGF-2 supplementation are limited to early passages of adipocyte precursors, while sustained supplementation in later passages is associated with detrimental effects due to the decline in FGFR1 expression and subsequent reduction in STAT3 phosphorylation [121]. In human adipocytes, FGF-2 and FGFR1 are downregulated as they differentiate into adipocytes. Adipocyte precursors expressing a dominant negative form of FGFR1 or treated with a specific inhibitor of FGFR1 signaling completely lose their ability to form lipid-containing adipocytes. Thus, FGF signaling plays a crucial role in both the expansion of progenitors and their subsequent differentiation [122].

Additional FGFs have been demonstrated to be either produced by or exert effects on adipocyte precursors, although the roles of specific FGFs in adipocyte precursor proliferation are limited. *Fgf10* is highly expressed in WAT, particularly in APs [123]. FGF10 acts on AP in WAT through autocrine/paracrine signaling, promoting cell proliferation via the activation of FGFR2b and the Ras/MAPK pathway [124]. WAT development is greatly impaired in *Fgf10* knockout mouse embryos, however, its roles at postnatal stages remain unclear as *Fgf10* knockout mice die shortly after birth with impaired multi-organ formation [123]. FGF6, a paracrine factor primarily expressed in fully differentiated adipocytes, stimulates the proliferation of PDGFRα+ adipocyte precursor cells through ERK signaling and is downregulated in mature adipocytes during obesity and aging [125]. Furthermore, mice subjected to a high-fat diet, with persistent inguinal WAT-specific FGF6 blockade using a neutralizing antibody or *Fgf6*-null mice, display notable adipocyte hypertrophy, adipose fibrosis, inflammation, and impaired glucose tolerance that is coupled with an expedited deficiency in adipocyte precursor abundance [125]. These results suggest that FGF6 plays a protective role in maintaining the adipocyte precursor pool to maintain metabolic health.

## BMP Signaling and Adipogenic Precursor Commitment

Perhaps, among growth factors, none have been as extensively examined for their involvement in adipogenesis as the bone morphogenetic proteins (BMPs). BMP2, BMP4, BMP7, BMP9, and others are associated with the direct control of ASCs and their commitment to adipocyte progenitors (APs). This regulatory influence can steer their differentiation toward white, beige, or brown adipocytes, depending on the specific BMP ligand or the context (Figure 1L) [126–129].

### BMP2 directs adipogenic precursors towards a white adipocyte fate

Experiments with mouse cell lines have shown that supplementing exogenous BMP2 promotes adipogenesis in 3T3L1 APs and induces commitment to the adipocyte lineage in C3H10T1/2 MSCs [126,130]. This response is orchestrated via SMAD1/5/8 signaling, resulting in increased expression of the adipogenic transcription factors PPARγ and C/EBPα [131,132]. Human abdominal and gluteal adipose tissue, along with adipogenic precursors isolated from these tissues, exhibit BMP2 expression [133]. In human abdominal adipocyte precursors, BMP2 signaling includes SMAD1/5/8 phosphorylation, leading to enhanced PPARγ expression and triacylglyceride accumulation [133]. Intriguingly, multiple studies have revealed an association between a BMP2-linked polymorphism (rs979012) and an increased waist-to-hip ratio and BMI, underscoring the role of BMP signaling in adipose tissue physiology [133–135].

### BMP4 induces a white or brown phenotype based on the cellular context

Previous research on BMP4 revealed its capacity to commit C3H10T1/2 MSCs to an adipocyte lineage, as indicated by an increased occurrence of adipocyte formation when exposed to inducers of adipogenic differentiation [126,127,136]. Adipocytes in mice, featuring transgenic overexpression of BMP4 under the *Fabp4* promoter, display browning of inguinal WAT, increased energy expenditure and protection against high-fat diet-induced obesity, coupled with enhanced insulin sensitivity [137]. Conversely, mice lacking *Bmp4* display hypertrophy of white adipocytes and heightened insulin resistance [137]. Intriguingly, the expression of BMP4 in human WAT shows a negative correlation with body mass index, indicating its potential role in promoting increased energy expenditure [137].

The role of BMP4 in determining a white or beige/brown phenotype is cell type-dependent. BMP4 induces MSCs to commit to APs, promoting beige adipogenesis. However, in mature adipocytes, BMP4 suppresses the beige phenotype and supports the development of a white phenotype [128]. BMP4 is present in both APs and mature adipocytes, yet in WAT APs, the potential of BMP4 to stimulate beige adipogenesis might be inhibited by the expression of gremlin 1 (GREM1), an inhibitor of BMP4/7 [138,139]. BMP4 expression is evident in brown preadipocytes, as well as other cells within the SVF, including MSCs and endothelial cells, with a gradual decrease observed during the final stages of brown adipocyte differentiation [140]. In contrast, white adipocytes exhibit higher BMP4 expression in mature adipocytes compared to the SVF [141]. Hence, it has been suggested that BMP4 signaling gradually diminishes from the stem cell niche to the mature adipocyte during the development of beige and brown adipocytes, possibly attributed to local diffusion and the synthesis of BMP4 antagonists [140].

The control of beige and brown adipogenesis by BMP4 derived from adipose tissue may also involve its impact on other cells present in the adipose tissue microenvironment. In a recent investigation, *Fabp4*-*Bmp4* transgenic mice were shown to stimulate the proliferation of CD206+ M2 macrophages while inhibiting M1 macrophages, resulting in a substantial rise in M2 macrophage numbers [141]. This phenomenon might explain the observed enhancement of WAT browning. Consistent with this notion, the transfer of BMP4-induced M2 macrophages to subcutaneous WAT resulted in the upregulation of brown adipocyte markers, including PRDM16, peroxisome proliferator-activated receptor-gamma coactivator 1 alpha (PGC1α), and UCP1, accompanied by an elevation in whole-body oxygen consumption [141]. Therefore, the findings from earlier investigations indicating that BMP4 induces a browning effect in WAT, leading to heightened energy expenditure and improved metabolism, might be attributed, at least in part, to the regulatory role of BMP4 on macrophages (Figures 1J and 2E).

### BMP7 is necessary for brown/beige adipocyte precursor commitment

Investigations using *Bmp7* null mice have demonstrated the essential role of BMP7 in the development and differentiation of brown adipocyte precursors, leading to the formation of brown adipose tissue and preservation of its thermogenic program [129]. Notably, administering BMP7 systemically to mice effectively counteracts obesity by enhancing energy expenditure and suppressing appetite [142]. Genetic deletion of the type 1A BMP receptor (*Bmpr1a*) within the MYF5+ lineage, the embryonic precursor cells to brown adipose tissue [1], results in a deficiency of interscapular BAT and stimulates beige adipogenesis in WAT [143]. This effect, mediated by heightened sympathetic nervous system input, indicates intercommunication between these two adipose depots and reinstates the overall thermogenic capacity mediated by thermogenic adipocytes, ensuring the preservation of normal body temperature and resistance to diet-induced obesity [143]. Adipogenic precursors obtained from subcutaneous WAT exhibit synergistic induction toward a BAT-like phenotype when treated with BMP7 together with β3-adrenergic agonists. This implies a direct involvement of BMP signaling in promoting beige adipogenesis [144,145]. BMP7 has also been utilized in the *ex vivo* generation of brown adipocytes from both human embryonic stem cells and iPSCs, highlighting its potential significance in the development of human BAT [146].

The signaling and transcriptional mechanisms underlying BMP regulation of adipogenesis are not fully understood. However, there is evidence suggesting that BMP7 facilitates brown and beige adipogenesis by regulating EBF2 and ZFP423 via SMAD-induced mechanisms [147]. EBF2 serves as a transcription factor with selective expression in brown and beige adipocyte precursors, playing a regulatory role in the expression of target genes specific to brown adipose tissue, including *Prdm16* [148–150]. The transcriptional regulator ZFP423 acts as a corepressor of EBF2, playing a crucial role in preserving the identity of white adipocytes by suppressing *Prdm16* [151]. Upon activation of adipocyte precursors by BMP7, SMAD1/4 engages with ZFP423, leading to the disruption of the ZFP423-EBF2 protein complex. This interaction facilitates EBF2 to initiate the expression of target genes associated with brown adipogenesis, including *Prdm16* [151].

### BMP9 is a secreted hepatokine that initiates browning of WAT adipogenic precursors

BMP-9 is a hepatokine that regulates glucose homeostasis-related enzymes and injection of recombinant human BMP-9 successfully reduces blood glucose levels in diabetic mice [152]. Furthermore, recombinant BMP9 promotes brown adipogenesis in human ASCs [153]. Intraperitoneal administration of BMP9 in mice with high-fat diet-induced obesity results in significant browning of subcutaneous WAT, leading to reduced weight gain, smaller white adipocytes and decreased fasting blood glucose levels [153]. Cold exposure in mice for 3 weeks increases hepatic BMP9 expression and plasma levels [154]. Treatment of cell cultures with BMP9 facilitates the differentiation of subcutaneous WAT-derived adipogenic precursors into beige adipocytes. This is evidenced by elevated expression levels of markers associated with brown adipocytes and mitochondrial biogenesis, including increased UCP1 and PGC1α expression, respectively [154]. *In vivo* administration of BMP9 triggers the expression of browning markers in WAT. In mice fed a high-fat diet, BMP9 administration protects against obesity and improves glucose tolerance [154]. In conclusion, either pharmacological treatment with BMP9 or liver secretion of BMP9 induced by cold exposure can promote adipocyte browning, leading to a reduction in fat mass and amelioration of dysregulated blood glucose levels in high-fat diet-induced obese mice.

## Regulation of ASC Pluripotency and Self-renewal

Ensuring the continual self-renewal and multipotency potential within the ASC pool is a crucial element for the overall regulation and equilibrium of adipose tissue homeostasis. This dynamic equilibrium is essential for the proper functioning of adipose tissue, influencing aspects such as adipocyte turnover, tissue repair, and responsiveness to physiological demands [155]. OCT4, SOX2, and NANOG are transcription factors that inhibit genes associated with differentiation, preserving pluripotency [156,157]. Through physical interactions, they modulate each other’s expression, thereby regulating target genes crucial for self-renewal and pluripotency [156,157]. While OCT4, NANOG, and SOX2 are expressed at lower levels in ASCs compared to embryonic stem cells, several studies have consistently shown that these genes are linked to heightened self-renewal and multipotency within the ASC population (Figure 1L) [30,158,159]. Like CD34, a widely recognized marker for ASCs that undergoes rapid downregulation during *ex vivo* expansion, the levels of OCT4, NANOG, and SOX2 also diminish rapidly in cell culture [160]. This rapid decrease in expression may explain previous controversy surrounding the expression of these markers in ASCs observed in earlier studies. In support of this, isolation techniques that enhance the purity of primary CD34+ ASCs lead to a 2–3 fold increase in the expression of OCT4, NANOG, and SOX2 [161]. During replicative senescence associated with extended *ex vivo* expansion of ASCs, heightened levels of reactive oxygen species (ROS) lead to diminished proliferation, pluripotency, and expression of OCT4, NANOG, and SOX2 [162]. This senescent state is concomitant with ROS-mediated reduction of the transcription factor c-MAF, known for its direct binding to and regulation of OCT4, NANOG, and SOX2 expression [162]. Lastly, elements that enhance pluripotency in mouse embryonic stem cells, such as the addition of leukemia inhibitory factor (LIF) or the overexpression of the mir-302 cluster, also contribute to the capacity of ASCs to sustain the expression of pluripotency genes [160].

Functional investigations have revealed the involvement of pluripotency genes in governing ASC functionality. Elevated OCT4 expression leads to the demethylation of regulatory regions associated with stemness genes, including OCT4, NANOG, and SOX2, thereby enhancing ASC proliferation and multipotency [163]. The suppression of NANOG in ASCs induces a reduction in the expression of OCT4 and SOX2 genes, leading to decreased proliferation attributed to cell cycle arrest in G0/G1 [164]. Within MSCs derived from bone marrow, OCT4 and NANOG exhibit binding activity to the promoter region of DNA methyltransferase 1 (DNMT1) [165]. This interaction plays a crucial role in suppressing genes associated with differentiation by maintaining methylation levels during DNA replication. Consistent with this, the inhibition of NANOG in ASCs results in a decline in pluripotency and differentiation capacity through the downregulation of DNMT1 [166–168].

The precise regulation of *Nanog* and *Oct4* expression in ASCs might be partially influenced by programmed cell death 4 (*Pdcd4*), a protein translation suppressor linked to diet-induced obesity, WAT inflammation and insulin resistance [155,169]. *Pdcd4* ablation in mice correlates with elevated levels of *Oct4* and *Nanog*, leading to heightened stemness and proliferation of ASCs through increased AKT activation and upregulation of cyclinD1 [155]. Remarkably, *Pdcd4* ablation also promotes the transition from white to beige adipocytes, resulting in augmented energy expenditure and resistance to obesity on a high-fat diet [155]. These findings imply that enhanced stemness of ASCs could play a beneficial role in preventing obesity and metabolic syndrome.

Genes specifically associated with specific functions related to adipose tissue might also govern the expression of pluripotency genes in ASCs. As the proliferation and differentiation potential of ASCs decline with successive passages in culture, there is a simultaneous decrease in PPARγ and thyroid hormone receptor (TRβ), concomitant with the loss of OCT4 [170]. Administering PPARγ agonists to ASCs enhances OCT4 promoter activity, expression, and rejuvenates differentiation potential as ASCs undergo aging in culture [170]. Throughout the culture of ASCs, the observed decline in proliferative potential and stemness is additionally linked to an age-related reduction in proteasome complex and peptidase activities [171]. In particular, the activation of the proteasome is correlated with reduced levels of ROS, elongated telomeres, and elevated expression of OCT4, NANOG, and SOX2, simultaneously leading to an augmentation in stemness [171]. Conversely, the silencing of OCT4 or NANOG leads to a notable decrease in proteasomal activity, attributed to the diminished association of OCT4 with β2 and β5 proteasomal subunit promoters, indicating that pluripotency genes might, in part, govern stemness through modulation of proteostasis [171]. Overall, the intricate interplay between pluripotency genes and various factors highlights the complexity of ASC regulation, offering insights into potential strategies for manipulating their cellular properties.

## Impaired Functions of Adipocyte Precursors in Obesity and Metabolic Disease

When exposed to a high-fat diet, adipocyte precursors contribute to the onset of adipocyte hyperplasia. However, the extent of new adipocyte formation varies among distinct adipose tissue depots [172]. In mice, the initiation of adipogenesis in the visceral WAT depot occurs significantly before adipocyte hypertrophy that occurs during the development of diet-induced obesity [65]. Likewise, the enlargement of human visceral WAT may rely on a surge in adipocyte proliferation during the initiation of obesity [173]. Notably, both in humans and mice, fully developed obesity is linked to a decline in adipocyte precursors and a diminished capacity for their differentiation, which can result in adipocyte hypertrophy, adipose dysfunction, and the onset of metabolic syndrome [174,175]. Hence, during periods of energy excess and the onset of diabetes, adipocyte precursors exhibit efficient proliferation and differentiation into adipocytes, yet face impairment in later stages, potentially contributing to adipocyte hypertrophy and the exacerbation of metabolic abnormalities [30]. Moreover, in individuals with obesity, a reduction in ASC activity may constrain their overall multipotent and regenerative capacity, which are critical factors for patients who could potentially benefit from autologous transplantation.

In contrast to lean individuals, ASCs extracted from subcutaneous and visceral WAT in obese patients exhibit decreased cell proliferation, premature senescence, and a decline in both angiogenic potential and the ability for multilineage differentiation [91,174,176–178]. ASCs in obese patients demonstrate a compromised capacity to release proangiogenic factors such as VEGF, HGF, FGF, and PDGF. This impairment can hinder angiogenesis, induce hypoxia, and lead to cellular stress, ultimately contributing to adipocyte death [30,132–135]. In obese individuals, the decline in ASC multipotency is associated with an upregulation of genes related to inflammation and a concurrent downregulation of genes associated with embryonic development and multilineage differentiation, including TBX15, HOXC10, and α-SMA, potentially contributing to a compromised tissue repair capacity [91,177].

ASCs have been observed to exhibit robust immunosuppressive activity, playing a crucial role in the regulation of inflammation and immunopathologic responses. Additionally, this characteristic is significant for the development and application of immunomodulation therapies and allogeneic stem cell treatments [179,180]. ASCs obtained from obese and type 2 diabetic individuals exhibit diminished immunosuppressive functions, such as suppressing immune cell proliferation and polarizing macrophages towards the M2 phenotype, in contrast to those derived from lean individuals (Figure 2F) [181]. ASCs from obese individuals exhibit up-regulation of inflammatory genes such as IL-1β, IL-8, and monocyte chemoattractant protein 1 (MCP1), previously associated with elevated BMI and linked to cardiovascular disease and type 2 diabetes risk [91]. The increased expression of these cytokines, coupled with elevated levels of tumor necrosis factor-α (TNFα) and IL-6 in ASCs, may potentially contribute to the initiation or exacerbation of adipose tissue inflammation and foster insulin resistance by attracting and directing macrophages towards the M1 subtype (Figure 2F) [30,182–185].

Genes associated with stemness exhibit dysregulation in obesity and type 2 diabetes, with ASCs from subcutaneous WAT of obese patients displaying decreased expression of OCT4, SAL4, SOX15, and KLF4, while ASCs from omental WAT show increased expression [186]. In a separate investigation, heightened expression of OCT4 and NANOG was noted in ASCs obtained from both subcutaneous and visceral WAT in diabetic individuals compared to those without diabetes [187]. Additional markers of stemness, such as reduced viability of ASCs in cell culture, diminished telomerase activity, and shortened telomere length, provide further evidence of the disrupted self-renewal capacity in ASCs derived from individuals with obesity [188]. Concerning telomerase activity, the selective disruption of either *Pdgfrα* + or *Pdgfrβ*+ adipocyte progenitor lineages in telomerase reverse transcriptase gene knockout mice results in premature telomere shortening, adipocyte progenitor senescence, and subsequent adverse metabolic effects. These effects include adipocyte hypertrophy, inflammation, and fibrosis in adipose tissue, as well as systemic insulin resistance, further exacerbated by a high-fat diet [189]. This information suggests that excessive nutrient intake can induce senescence in adipose progenitor cells, establishing a mechanistic connection between aging, obesity, and diabetes.

In obese individuals, inflammatory cytokines like IL-6 or TNFα are known to shorten cilia in ASCs, impairing their ability to respond adequately to stimuli [190]. The use of an inhibitor targeting Aurora A, a kinase involved in cilia disassembly, has been shown to reverse this phenotype, restoring cilia length and function in obese ASCs [191]. Remarkably, this reversal is linked to an upregulation of self-renewal and stemness genes, suggesting a potential approach to address obesity-related disorders [191].

Overall, disturbances in gene networks associated with stemness, inflammation, multilineage potential, and ASC trafficking and homing contribute to ASC dysfunction [91]. Dysfunctional ASCs, by disrupting adipose tissue remodeling, promoting inflammation, and inducing hypoxia, may play a role in the development of obesity and related diseases [30,192]. Supporting this notion, supplementing obese mice with ASCs from lean counterparts has been found to reduce adipose inflammation, enhance insulin action, and restore metabolic balance [175]. Therefore, ensuring effective proliferation, renewal, and differentiation of ASCs is crucial for proper adipose tissue function, and the restoration of ASCs could be a promising strategy in combating obesity-related diseases [30,155].

## Lifestyle Interventions in Adipose Tissue Remodeling

Adipose tissue exhibits remarkable adaptability in response to changes in energy demand. Exercise plays a pivotal role in modulating the endocrine profile of adipose tissues, regulating mitochondrial activity, and enhancing glucose uptake [193]. The enlargement and reduced efficiency of adipocytes seen in obesity are often linked to increased lipogenesis and impaired angiogenesis. It’s widely acknowledged that exercise upregulates *Mdm2* expression, promoting angiogenesis, stimulating lipolysis, and suppressing lipogenic gene expression in adipose tissue [194–196]. These mechanisms, which counteract hypoxia and hypertrophic adipocyte development, likely contribute to the promotion of smaller, metabolically healthier adipocytes through exercise.

Adipose tissue harbors functionally distinct populations of APs identified by CD34 and CD9 as pro-adipogenic (CD34^low^ and CD9^low^) and pro-fibrotic (CD34^high^ and CD9^high^] [197–200]. Recent research suggests intense exercise reduces pro-fibrotic CD34^high^ adipocyte precursors in human abdominal subcutaneous fat without affecting CD34^low^ APs in the same individuals, indicating exercise’s potential to favorably modulate the adipocyte precursor pool [201]. Animal studies have shown that exercise training increases vascular density in the adipose tissue of obese rats compared to sedentary counterparts [202]. Similarly, clinical investigations involving individuals with insulin resistance demonstrate that both sprint interval training and moderate-intensity continuous training elevate vascular density in subcutaneous adipose tissue [203]. Exercise’s immunomodulatory effects may also mitigate obesity-induced inflammation in adipose tissue by reducing inflammatory macrophages and promoting an anti-inflammatory M2 macrophage phenotype [202,204,205]. Furthermore, exercise decreases the number of CD8^+^ T cells and macrophage infiltration in obesity-related adipose tissue, while enhancing the secretion of anti-inflammatory cytokines IL6 and IL10 and shifting macrophage phenotype from M1 to M2 in the subcutaneous fat of high-fat diet mice, resulting in improved insulin sensitivity [205].

In rodents, calorie restriction decreases fat mass, delays age-related diseases like type 2 diabetes, and extends lifespan [206]. Similarly, in obese individuals, calorie restriction, weight loss, and exercise enhance insulin sensitivity [207]. Studies by Fabbiano *et al*. indicate that calorie restriction reduces total fat mass while promoting the browning of white adipose tissue, accompanied by increased eosinophils, M2 macrophages, and type 2 cytokine signaling [208]. Calorie restriction stimulates mitochondrial biogenesis in various tissues, including skeletal muscle, liver, heart, and WAT, with SREBP-1c mediating mitochondrial biogenesis specifically in WAT [209–211]. The correlation between calorie restriction and browning markers in humans remains ambiguous, as studies have demonstrated that observed improvements in body fat and insulin resistance appear to be unrelated to browning [212].

## Conclusions and Future Perspectives

Understanding the adipose tissue stem cell niche has been hindered by challenges in identifying specific markers for distinct cell subpopulations, the absence of optimal cell culture conditions maintaining cells in their native state, and a lack of *in vivo* models for monitoring cells within the native niche. Recent developments in 3D culture techniques and the discovery of new adipogenic growth factors offer potential solutions, allowing for the preservation and more accurate representation of the native niche *ex vivo*. This is particularly crucial when studying the physiological abnormalities of adipogenic precursors from obese patients, as culture conditions may inaccurately reflect the native niche, leading to incorrect assumptions about their role in disease progression. Supporting this, optimized 3D culture conditions that maintain the niche environment have already demonstrated improved clinical effects [213].

Earlier research has proposed that various cell types within the niche (such as endothelial, hematopoietic, mural, and fibroblastic cells) have the potential to serve as adipocyte precursors *in vitro*. However, lineage tracing studies in mice are now providing insights into the key populations contributing to mature adipocytes during normal metabolic homeostasis, weight gain, and WAT browning. Specifically, a fibroblast stem cell population, identified by PDGFRα and the mural cell marker PDGFRβ, seems to play a significant role in the formation of the majority of mature adipocytes in adult mice [82]. Advances in single-cell RNA sequencing and the identification of non-cell surface markers for tracking and purifying adipogenic precursors may enable a more precise categorization of these cells into an ordered hierarchy of adipogenic commitment and potential.

ASCs and progenitors obtained from subcutaneous and visceral adipose tissues of obese individuals exhibit diminished cell proliferation, premature senescence, and a decline in angiogenic potential, as well as the capacity for multilineage differentiation [91,174–178]. Consequently, in the later stages of obesity, the impaired function of ASCs and adipocyte progenitors may contribute to adipocyte hypertrophy linked to excessive caloric intake and insulin resistance. Additionally, ASCs derived from obese and type 2 diabetes patients exhibit reduced immunosuppressive activity, including the inhibition of immune cell proliferation and the ability to polarize macrophages toward the M2 phenotype [181]. Inflammatory cytokines linked to cardiovascular disease and type 2 diabetes risk are also upregulated in ASCs [91]. Collectively, the restoration of ASCs through transplantation emerges as a potentially effective strategy to address complications associated with obesity, as demonstrated in mouse studies [175].

Understanding the molecular mechanisms governing the self-renewal and differentiation of ASCs and their downstream progenitors holds promise for the development of innovative therapies to address obesity-related disorders. For instance, exploring the mechanisms linked to BMP7-induced differentiation of beige/brown adipocytes may offer insights into developing therapies that enhance thermogenesis and provide protection against metabolic syndrome. There is a growing awareness of the significant impact macrophages have on both the renewal and differentiation potential of adipogenic precursors, with M2 macrophage subsets playing a role in maintaining a healthy balance between renewal and differentiation [111]. Identifying factors associated with polarization toward the M2 phenotype (such as BMP4/IL-4) or factors from these cells that directly promote the generation of beige adipocytes from adipocyte precursors may open avenues for developing novel therapies for weight loss [109,110].

Creating conditions conducive to the functional co-culture of various cell types within the adipose precursor niche is increasingly crucial for advancing our understanding of how these cells interact and influence adipogenesis during normal, insulin-resistant and inflammatory states. The successful establishment of co-culture methods involving different subpopulations of ASCs, progenitors, macrophages, endothelial cells, neurons, mural cells, fibroblasts, and other cell types is paramount. For instance, attempts to co-culture sympathetic neurons with ASCs for studying lipolysis and adipose tissue browning have faced challenges, potentially attributed to the secretion of factors in co-culture that hinder differentiation when the cells are isolated outside the native adipose tissue niche [214]. Moreover, the research community is actively exploring the therapeutic potential of ASCs across various diseases and regenerative applications, encompassing soft tissue defect reconstruction, wound healing, skin restoration, skeletal muscle/bone reconstruction, liver regeneration and cardiac repair [215]. Future identification of new regulatory factors governing the adipocyte precursor niche will contribute to determining optimal culturing methods for ASCs. This is not only crucial for advancing the study of metabolic diseases, but also for harnessing the potential of these cells in the field of regenerative medicine.

## References

[1] Harms M, Seale P. Brown and beige fat: development, function and therapeutic potential. Nat Med. 2013;19(10):1252–63. 24100998 10.1038/nm.3361

[2] National Diabetes Statistics Report website. https://www.cdc.gov/diabetes/data/statistics-report/index.html. Centers for Disease Control and Prevention. Accessed, May 2024.

[3] Malik VS, Willett WC, Hu FB. Global obesity: trends, risk factors and policy implications. Nat Rev Endocrinol. 2013;9(1):13–27. 23165161 10.1038/nrendo.2012.199

[4] Ochner CN, Tsai AG, Kushner RF, Wadden TA. Treating obesity seriously: when recommendations for lifestyle change confront biological adaptations. Lancet Diabetes Endocrinol. 2015;3(4):232–4. 25682354 10.1016/S2213-8587(15)00009-1

[5] Lumeng CN, Saltiel AR. Inflammatory links between obesity and metabolic disease. J Clin Invest. 2011;121(6):2111–7. 21633179 10.1172/JCI57132 PMC3104776

[6] Fuster JJ, Ouchi N, Gokce N, Walsh K. Obesity-Induced Changes in Adipose Tissue Microenvironment and Their Impact on Cardiovascular Disease. Circ Res. 2016;118(11):1786–807. 27230642 10.1161/CIRCRESAHA.115.306885 PMC4887147

[7] Cildir G, Akincilar SC, Tergaonkar V. Chronic adipose tissue inflammation: all immune cells on the stage. Trends Mol Med. 2013;19(8):487–500. 23746697 10.1016/j.molmed.2013.05.001

[8] Chawla A, Nguyen KD, Goh YP. Macrophage-mediated inflammation in metabolic disease. Nat Rev Immunol. 2011;11(11):738–49. 21984069 10.1038/nri3071 PMC3383854

[9] Weisberg SP, McCann D, Desai M, Rosenbaum M, Leibel RL, Ferrante AW, Jr. Obesity is associated with macrophage accumulation in adipose tissue. J Clin Invest. 2003;112(12):1796–808. 14679176 10.1172/JCI19246 PMC296995

[10] Patsouris D, Li PP, Thapar D, Chapman J, Olefsky JM, Neels JG. Ablation of CD11c-positive cells normalizes insulin sensitivity in obese insulin resistant animals. Cell Metab. 2008;8(4):301–9. 18840360 10.1016/j.cmet.2008.08.015 PMC2630775

[11] Wentworth JM, Naselli G, Brown WA, Doyle L, Phipson B, Smyth GK, Pro-inflammatory CD11c+CD206+ adipose tissue macrophages are associated with insulin resistance in human obesity. Diabetes. 2010;59(7):1648–56. 20357360 10.2337/db09-0287 PMC2889764

[12] Cipolletta D, Feuerer M, Li A, Kamei N, Lee J, Shoelson SE, PPAR-gamma is a major driver of the accumulation and phenotype of adipose tissue Treg cells. Nature. 2012;486(7404):549–53. 22722857 10.1038/nature11132 PMC3387339

[13] Feuerer M, Herrero L, Cipolletta D, Naaz A, Wong J, Nayer A, Lean, but not obese, fat is enriched for a unique population of regulatory T cells that affect metabolic parameters. Nat Med. 2009;15(8):930–9. 19633656 10.1038/nm.2002 PMC3115752

[14] Nishimura S, Manabe I, Nagasaki M, Eto K, Yamashita H, Ohsugi M, CD8+ effector T cells contribute to macrophage recruitment and adipose tissue inflammation in obesity. Nat Med. 2009;15(8):914–20. 19633658 10.1038/nm.1964

[15] Wu D, Molofsky AB, Liang HE, Ricardo-Gonzalez RR, Jouihan HA, Bando JK, Eosinophils sustain adipose alternatively activated macrophages associated with glucose homeostasis. Science. 2011;332(6026):243–7. 21436399 10.1126/science.1201475 PMC3144160

[16] Sun K, Wernstedt Asterholm I, Kusminski CM, Bueno AC, Wang ZV, Pollard JW, Dichotomous effects of VEGF-A on adipose tissue dysfunction. Proc Natl Acad Sci U S A. 2012;109(15):5874–9. 22451920 10.1073/pnas.1200447109 PMC3326476

[17] Sung HK, Doh KO, Son JE, Park JG, Bae Y, Choi S, Adipose vascular endothelial growth factor regulates metabolic homeostasis through angiogenesis. Cell Metab. 2013;17(1):61–72. 23312284 10.1016/j.cmet.2012.12.010

[18] Hajer GR, van Haeften TW, Visseren FL. Adipose tissue dysfunction in obesity, diabetes, and vascular diseases. Eur Heart J. 2008;29(24):2959–71. 18775919 10.1093/eurheartj/ehn387

[19] Ruan H, Hacohen N, Golub TR, Van Parijs L, Lodish HF. Tumor necrosis factor-alpha suppresses adipocyte-specific genes and activates expression of preadipocyte genes in 3T3-L1 adipocytes: nuclear factor-kappaB activation by TNF-alpha is obligatory. Diabetes. 2002;51(5):1319–36. 11978627 10.2337/diabetes.51.5.1319

[20] Qiao L, Zou C, van der Westhuyzen DR, Shao J. Adiponectin reduces plasma triglyceride by increasing VLDL triglyceride catabolism. Diabetes. 2008;57(7):1824–33. 18375436 10.2337/db07-0435 PMC2453618

[21] Hopkins TA, Ouchi N, Shibata R, Walsh K. Adiponectin actions in the cardiovascular system. Cardiovasc Res. 2007;74(1):11–8. 17140553 10.1016/j.cardiores.2006.10.009 PMC1858678

[22] Aldhahi W, Hamdy O. Adipokines, inflammation, and the endothelium in diabetes. Curr Diab Rep. 2003;3(4):293–8. 12866991 10.1007/s11892-003-0020-2

[23] Matsuzawa Y The metabolic syndrome and adipocytokines. FEBS Lett. 2006;580(12):2917–21. 16674947 10.1016/j.febslet.2006.04.028

[24] Verma S, Li SH, Wang CH, Fedak PW, Li RK, Weisel RD, Resistin promotes endothelial cell activation: further evidence of adipokine endothelial interaction. Circulation. 2003;108(6):736–40. 12874180 10.1161/01.CIR.0000084503.91330.49

[25] Calabro P, Samudio I, Willerson JT, Yeh ET. Resistin promotes smooth muscle cell proliferation through activation of extracellular signal-regulated kinase 1/2 and phosphatidylinositol 3-kinase pathways. Circulation. 2004;110(21):3335–40. 15545519 10.1161/01.CIR.0000147825.97879.E7

[26] Domouzoglou EM, Naka KK, Vlahos AP, Papafaklis MI, Michalis LK, Tsatsoulis A, Fibroblast growth factors in cardiovascular disease: The emerging role of FGF21. Am J Physiol Heart Circ Physiol. 2015;309(6):H1029–38. 26232236 10.1152/ajpheart.00527.2015 PMC4747916

[27] Branen L, Hovgaard L, Nitulescu M, Bengtsson E, Nilsson J, Jovinge S. Inhibition of tumor necrosis factor-alpha reduces atherosclerosis in apolipoprotein E knockout mice. Arterioscler Thromb Vasc Biol. 2004;24(11):2137–42. 15345516 10.1161/01.ATV.0000143933.20616.1b

[28] Rosen ED, Spiegelman BM. What we talk about when we talk about fat. Cell. 2014;156(1–2):20–44. 24439368 10.1016/j.cell.2013.12.012 PMC3934003

[29] Cannon B, Nedergaard J. Brown adipose tissue: function and physiological significance. Physiol Rev. 2004;84(1):277–359. 14715917 10.1152/physrev.00015.2003

[30] Louwen F, Ritter A, Kreis NN, Yuan J. Insight into the development of obesity: functional alterations of adipose-derived mesenchymal stem cells. Obes Rev. 2018;19(7):888–904. 29521029 10.1111/obr.12679

[31] Cawthorn WP, Scheller EL, MacDougald OA. Adipose tissue stem cells meet preadipocyte commitment: going back to the future. J Lipid Res. 2012;53(2):227–46. 22140268 10.1194/jlr.R021089 PMC3269153

[32] Ramakrishnan VM, Boyd NL. The Adipose Stromal Vascular Fraction as a Complex Cellular Source for Tissue Engineering Applications. Tissue Eng Part B Rev. 2018;24(4):289–99. 28316259 10.1089/ten.teb.2017.0061 PMC6080106

[33] Seaberg RM, van der Kooy D. Stem and progenitor cells: the premature desertion of rigorous definitions. Trends Neurosci. 2003;26(3):125–31. 12591214 10.1016/S0166-2236(03)00031-6

[34] Rodeheffer MS, Birsoy K, Friedman JM. Identification of white adipocyte progenitor cells in vivo. Cell. 2008;135(2):240–9. 18835024 10.1016/j.cell.2008.09.036

[35] Berry R, Rodeheffer MS, Rosen CJ, Horowitz MC. Adipose Tissue Residing Progenitors (Adipocyte Lineage Progenitors and Adipose Derived Stem Cells (ADSC). Curr Mol Biol Rep. 2015;1(3):101–9. 26526875 10.1007/s40610-015-0018-y PMC4624461

[36] Lee YH, Petkova AP, Mottillo EP, Granneman JG. In vivo identification of bipotential adipocyte progenitors recruited by beta3-adrenoceptor activation and high-fat feeding. Cell Metab. 2012;15(4):480–91. 22482730 10.1016/j.cmet.2012.03.009 PMC3322390

[37] Tang W, Zeve D, Suh JM, Bosnakovski D, Kyba M, Hammer RE, White fat progenitor cells reside in the adipose vasculature. Science. 2008;322(5901):583–6. 18801968 10.1126/science.1156232 PMC2597101

[38] Lee MJ, Wu Y, Fried SK. Adipose tissue heterogeneity: implication of depot differences in adipose tissue for obesity complications. Mol Aspects Med. 2013;34(1):1–11. 23068073 10.1016/j.mam.2012.10.001 PMC3549425

[39] Karpe F, Pinnick KE. Biology of upper-body and lower-body adipose tissue--link to whole-body phenotypes. Nat Rev Endocrinol. 2015;11(2):90–100. 25365922 10.1038/nrendo.2014.185

[40] Lee MJ, Wu Y, Fried SK. Adipose tissue remodeling in pathophysiology of obesity. Curr Opin Clin Nutr Metab Care. 2010;13(4):371–6. 20531178 10.1097/MCO.0b013e32833aabef PMC3235038

[41] Spalding KL, Arner E, Westermark PO, Bernard S, Buchholz BA, Bergmann O, Dynamics of fat cell turnover in humans. Nature. 2008;453(7196):783–7. 18454136 10.1038/nature06902

[42] Rigamonti A, Brennand K, Lau F, Cowan CA. Rapid cellular turnover in adipose tissue. PLoS One. 2011;6(3):e17637. 21407813 10.1371/journal.pone.0017637 PMC3047582

[43] Rosen ED, MacDougald OA. Adipocyte differentiation from the inside out. Nat Rev Mol Cell Biol. 2006;7(12):885–96. 17139329 10.1038/nrm2066

[44] Christodoulides C, Lagathu C, Sethi JK, Vidal-Puig A. Adipogenesis and WNT signalling. Trends Endocrinol Metab. 2009;20(1):16–24. 19008118 10.1016/j.tem.2008.09.002 PMC4304002

[45] Kim JB, Spiegelman BM. ADD1/SREBP1 promotes adipocyte differentiation and gene expression linked to fatty acid metabolism. Genes Dev. 1996;10(9):1096–107. 8654925 10.1101/gad.10.9.1096

[46] Payne VA, Au WS, Lowe CE, Rahman SM, Friedman JE, O’Rahilly S, C/EBP transcription factors regulate SREBP1c gene expression during adipogenesis. Biochem J. 2009;425(1):215–23. 19811452 10.1042/BJ20091112 PMC2913385

[47] Barak Y, Nelson MC, Ong ES, Jones YZ, Ruiz-Lozano P, Chien KR, PPAR gamma is required for placental, cardiac, and adipose tissue development. Mol Cell. 1999;4(4):585–95. 10549290 10.1016/s1097-2765(00)80209-9

[48] Rosen ED, Hsu CH, Wang X, Sakai S, Freeman MW, Gonzalez FJ, C/EBPalpha induces adipogenesis through PPARgamma: a unified pathway. Genes Dev. 2002;16(1):22–6. 11782441 10.1101/gad.948702 PMC155311

[49] Kliewer SA, Umesono K, Mangelsdorf DJ, Evans RM. Retinoid X receptor interacts with nuclear receptors in retinoic acid, thyroid hormone and vitamin D3 signalling. Nature. 1992;355(6359):446–9. 1310351 10.1038/355446a0 PMC6159885

[50] Ahmadian M, Suh JM, Hah N, Liddle C, Atkins AR, Downes M, PPARgamma signaling and metabolism: the good, the bad and the future. Nat Med. 2013;19(5):557–66. 23652116 10.1038/nm.3159 PMC3870016

[51] Ng CW, Poznanski WJ, Borowiecki M, Reimer G. Differences in growth in vitro of adipose cells from normal and obese patients. Nature. 1971;231(5303):445. 10.1038/231445a0 4931598

[52] Poznanski WJ, Waheed I, Van R. Human fat cell precursors. Morphologic and metabolic differentiation in culture. Lab Invest. 1973;29(5):570–6. 4753021

[53] Van RL, Bayliss CE, Roncari DA. Cytological and enzymological characterization of adult human adipocyte precursors in culture. J Clin Invest. 1976;58(3):699–704. 956396 10.1172/JCI108516 PMC333228

[54] Green H, Meuth M. An established pre-adipose cell line and its differentiation in culture. Cell. 1974;3(2):127–33. 4426090 10.1016/0092-8674(74)90116-0

[55] Green H, Kehinde O. An established preadipose cell line and its differentiation in culture. II. Factors affecting the adipose conversion. Cell. 1975;5(1):19–27. 165899 10.1016/0092-8674(75)90087-2

[56] Chawla A, Lazar MA. Peroxisome proliferator and retinoid signaling pathways co-regulate preadipocyte phenotype and survival. Proc Natl Acad Sci U S A. 1994;91(5):1786–90. 8127882 10.1073/pnas.91.5.1786 PMC43248

[57] Tontonoz P, Hu E, Spiegelman BM. Stimulation of adipogenesis in fibroblasts by PPAR gamma 2, a lipid-activated transcription factor. Cell. 1994;79(7):1147–56. 8001151 10.1016/0092-8674(94)90006-x

[58] Olson AL. RalA signaling may reveal the true nature of 3T3-L1 adipocytes as a model for thermogenic adipocytes. Proc Natl Acad Sci U S A. 2018;115(30):7651–3. 29976839 10.1073/pnas.1809686115 PMC6065013

[59] Klaus S, Choy L, Champigny O, Cassard-Doulcier AM, Ross S, Spiegelman B, Characterization of the novel brown adipocyte cell line HIB 1B. Adrenergic pathways involved in regulation of uncoupling protein gene expression. J Cell Sci. 1994;107 (Pt 1):313–9. 8175918 10.1242/jcs.107.1.313

[60] Galmozzi A, Sonne SB, Altshuler-Keylin S, Hasegawa Y, Shinoda K, Luijten IHN, ThermoMouse: an in vivo model to identify modulators of UCP1 expression in brown adipose tissue. Cell Rep. 2014;9(5):1584–93. 25466254 10.1016/j.celrep.2014.10.066 PMC4268417

[61] Sengenes C, Lolmede K, Zakaroff-Girard A, Busse R, Bouloumie A. Preadipocytes in the human subcutaneous adipose tissue display distinct features from the adult mesenchymal and hematopoietic stem cells. J Cell Physiol. 2005;205(1):114–22. 15880450 10.1002/jcp.20381

[62] Li H, Zimmerlin L, Marra KG, Donnenberg VS, Donnenberg AD, Rubin JP. Adipogenic potential of adipose stem cell subpopulations. Plast Reconstr Surg. 2011;128(3):663–72. 21572381 10.1097/PRS.0b013e318221db33 PMC4167367

[63] Mitchell JB, McIntosh K, Zvonic S, Garrett S, Floyd ZE, Kloster A, Immunophenotype of human adipose-derived cells: temporal changes in stromal-associated and stem cell-associated markers. Stem Cells. 2006;24(2):376–85. 16322640 10.1634/stemcells.2005-0234

[64] Hepler C, Vishvanath L, Gupta RK. Sorting out adipocyte precursors and their role in physiology and disease. Genes Dev. 2017;31(2):127–40. 28202540 10.1101/gad.293704.116 PMC5322728

[65] Jeffery E, Church CD, Holtrup B, Colman L, Rodeheffer MS. Rapid depot-specific activation of adipocyte precursor cells at the onset of obesity. Nat Cell Biol. 2015;17(4):376–85. 25730471 10.1038/ncb3122 PMC4380653

[66] Berry R, Rodeheffer MS. Characterization of the adipocyte cellular lineage in vivo. Nat Cell Biol. 2013;15(3):302–8. 23434825 10.1038/ncb2696 PMC3721064

[67] Napolitano L The Differentiation of White Adipose Cells. An Electron Microscope Study. J Cell Biol. 1963;18:663–79. 14064115 10.1083/jcb.18.3.663 PMC2106313

[68] Peurichard D, Delebecque F, Lorsignol A, Barreau C, Rouquette J, Descombes X, Simple mechanical cues could explain adipose tissue morphology. J Theor Biol. 2017;429:61–81. 28652001 10.1016/j.jtbi.2017.06.030

[69] Esteve D, Boulet N, Belles C, Zakaroff-Girard A, Decaunes P, Briot A, Lobular architecture of human adipose tissue defines the niche and fate of progenitor cells. Nat Commun. 2019;10(1):2549. 31186409 10.1038/s41467-019-09992-3 PMC6560121

[70] Iyama K, Ohzono K, Usuku G. Electron microscopical studies on the genesis of white adipocytes: differentiation of immature pericytes into adipocytes in transplanted preadipose tissue. Virchows Arch B Cell Pathol Incl Mol Pathol. 1979;31(2):143–55. 42211 10.1007/BF02889932

[71] Cinti S, Cigolini M, Bosello O, Bjorntorp P. A morphological study of the adipocyte precursor. J Submicrosc Cytol. 1984;16(2):243–51. 6325721

[72] Crisan M, Yap S, Casteilla L, Chen CW, Corselli M, Park TS, A perivascular origin for mesenchymal stem cells in multiple human organs. Cell Stem Cell. 2008;3(3):301–13. 18786417 10.1016/j.stem.2008.07.003

[73] Lazar MA. PPAR gamma, 10 years later. Biochimie. 2005;87(1):9–13. 15733730 10.1016/j.biochi.2004.10.021

[74] Vishvanath L, MacPherson KA, Hepler C, Wang QA, Shao M, Spurgin SB, Pdgfrbeta+ Mural Preadipocytes Contribute to Adipocyte Hyperplasia Induced by High-Fat-Diet Feeding and Prolonged Cold Exposure in Adult Mice. Cell Metab. 2016;23(2):350–9. 26626462 10.1016/j.cmet.2015.10.018 PMC4749445

[75] Long JZ, Svensson KJ, Tsai L, Zeng X, Roh HC, Kong X, A smooth muscle-like origin for beige adipocytes. Cell Metab. 2014;19(5):810–20. 24709624 10.1016/j.cmet.2014.03.025 PMC4052772

[76] Berry DC, Jiang Y, Graff JM. Mouse strains to study cold-inducible beige progenitors and beige adipocyte formation and function. Nat Commun. 2016;7:10184. 26729601 10.1038/ncomms10184 PMC4728429

[77] Su S, Guntur AR, Nguyen DC, Fakory SS, Doucette CC, Leech C, A Renewable Source of Human Beige Adipocytes for Development of Therapies to Treat Metabolic Syndrome. Cell Rep. 2018;25(11):3215–28 e9. 30540952 10.1016/j.celrep.2018.11.037 PMC6375695

[78] Brown AC. Brown adipocytes from induced pluripotent stem cells-how far have we come? Ann N Y Acad Sci. 2020;1463(1):9–22. 31573081 10.1111/nyas.14257 PMC7078043

[79] Berry DC, Jiang Y, Arpke RW, Close EL, Uchida A, Reading D, Cellular Aging Contributes to Failure of Cold-Induced Beige Adipocyte Formation in Old Mice and Humans. Cell Metab. 2017;25(1):166–81. 27889388 10.1016/j.cmet.2016.10.023 PMC5226893

[80] Li R, Bernau K, Sandbo N, Gu J, Preissl S, Sun X. Pdgfra marks a cellular lineage with distinct contributions to myofibroblasts in lung maturation and injury response. Elife. 2018;7:e36865. 30178747 10.7554/eLife.36865 PMC6122952

[81] Lee YH, Petkova AP, Konkar AA, Granneman JG. Cellular origins of cold-induced brown adipocytes in adult mice. FASEB J. 2015;29(1):286–99. 25392270 10.1096/fj.14-263038 PMC4285542

[82] Cattaneo P, Mukherjee D, Spinozzi S, Zhang L, Larcher V, Stallcup WB, Parallel Lineage-Tracing Studies Establish Fibroblasts as the Prevailing In Vivo Adipocyte Progenitor. Cell Rep. 2020;30(2):571–82 e2. 31940497 10.1016/j.celrep.2019.12.046

[83] Burl RB, Ramseyer VD, Rondini EA, Pique-Regi R, Lee YH, Granneman JG. Deconstructing Adipogenesis Induced by beta3-Adrenergic Receptor Activation with Single-Cell Expression Profiling. Cell Metab. 2018;28(2):300–9 e4. 29937373 10.1016/j.cmet.2018.05.025 PMC6082711

[84] Oguri Y, Shinoda K, Kim H, Alba DL, Bolus WR, Wang Q, CD81 Controls Beige Fat Progenitor Cell Growth and Energy Balance via FAK Signaling. Cell. 2020;182(3):563–77 e20. 32615086 10.1016/j.cell.2020.06.021 PMC7415677

[85] Coelho M, Oliveira T, Fernandes R. Biochemistry of adipose tissue: an endocrine organ. Arch Med Sci. 2013;9(2):191–200. 23671428 10.5114/aoms.2013.33181 PMC3648822

[86] Challa TD, Straub LG, Balaz M, Kiehlmann E, Donze O, Rudofsky G, Regulation of De Novo Adipocyte Differentiation Through Cross Talk Between Adipocytes and Preadipocytes. Diabetes. 2015;64(12):4075–87. 26340931 10.2337/db14-1932

[87] Zhang R, Gao Y, Zhao X, Gao M, Wu Y, Han Y, FSP1-positive fibroblasts are adipogenic niche and regulate adipose homeostasis. PLoS Biol. 2018;16(8):e2001493. 30080858 10.1371/journal.pbio.2001493 PMC6078284

[88] Nawaz A, Tobe K. M2-like macrophages serve as a niche for adipocyte progenitors in adipose tissue. J Diabetes Investig. 2019;10(6):1394–400. 10.1111/jdi.13114 PMC6825922 31293080

[89] Sun Y, Chen S, Zhang X, Pei M. Significance of Cellular Cross-Talk in Stromal Vascular Fraction of Adipose Tissue in Neovascularization. Arterioscler Thromb Vasc Biol. 2019;39(6):1034–44. 31018663 10.1161/ATVBAHA.119.312425 PMC6531320

[90] Bourin P, Bunnell BA, Casteilla L, Dominici M, Katz AJ, March KL, Stromal cells from the adipose tissue-derived stromal vascular fraction and culture expanded adipose tissue-derived stromal/stem cells: a joint statement of the International Federation for Adipose Therapeutics and Science (IFATS) and the International Society for Cellular Therapy (ISCT). Cytotherapy. 2013;15(6):641–8. 23570660 10.1016/j.jcyt.2013.02.006 PMC3979435

[91] Onate B, Vilahur G, Camino-Lopez S, Diez-Caballero A, Ballesta-Lopez C, Ybarra J, Stem cells isolated from adipose tissue of obese patients show changes in their transcriptomic profile that indicate loss in stemcellness and increased commitment to an adipocyte-like phenotype. BMC Genomics. 2013;14:625. 24040759 10.1186/1471-2164-14-625 PMC3848661

[92] Carey AL, Vorlander C, Reddy-Luthmoodoo M, Natoli AK, Formosa MF, Bertovic DA, Reduced UCP-1 content in in vitro differentiated beige/brite adipocytes derived from preadipocytes of human subcutaneous white adipose tissues in obesity. PLoS One. 2014;9(3):e91997. 24642703 10.1371/journal.pone.0091997 PMC3958417

[93] Kim WS, Han J, Hwang SJ, Sung JH. An update on niche composition, signaling and functional regulation of the adipose-derived stem cells. Expert Opin Biol Ther. 2014;14(8):1091–102. 24707959 10.1517/14712598.2014.907785

[94] Crandall DL, Hausman GJ, Kral JG. A review of the microcirculation of adipose tissue: anatomic, metabolic, and angiogenic perspectives. Microcirculation. 1997;4(2):211–32. 9219215 10.3109/10739689709146786

[95] Aoki S, Toda S, Sakemi T, Sugihara H. Coculture of endothelial cells and mature adipocytes actively promotes immature preadipocyte development in vitro. Cell Struct Funct. 2003;28(1):55–60. 12655151 10.1247/csf.28.55

[96] Traktuev DO, Prater DN, Merfeld-Clauss S, Sanjeevaiah AR, Saadatzadeh MR, Murphy M, Robust functional vascular network formation in vivo by cooperation of adipose progenitor and endothelial cells. Circ Res. 2009;104(12):1410–20. 19443841 10.1161/CIRCRESAHA.108.190926

[97] Traktuev DO, Merfeld-Clauss S, Li J, Kolonin M, Arap W, Pasqualini R, A population of multipotent CD34-positive adipose stromal cells share pericyte and mesenchymal surface markers, reside in a periendothelial location, and stabilize endothelial networks. Circ Res. 2008;102(1):77–85. 17967785 10.1161/CIRCRESAHA.107.159475

[98] Michailidou Z, Gomez-Salazar M, Alexaki VI. Innate Immune Cells in the Adipose Tissue in Health and Metabolic Disease. J Innate Immun. 2022;14(1):4–30. 33849008 10.1159/000515117 PMC8787575

[99] Russo L, Lumeng CN. Properties and functions of adipose tissue macrophages in obesity. Immunology. 2018;155(4):407–17. 30229891 10.1111/imm.13002 PMC6230999

[100] Lumeng CN, Bodzin JL, Saltiel AR. Obesity induces a phenotypic switch in adipose tissue macrophage polarization. J Clin Invest. 2007;117(1):175–84. 17200717 10.1172/JCI29881 PMC1716210

[101] Fujisaka S, Usui I, Bukhari A, Ikutani M, Oya T, Kanatani Y, Regulatory mechanisms for adipose tissue M1 and M2 macrophages in diet-induced obese mice. Diabetes. 2009;58(11):2574–82. 19690061 10.2337/db08-1475 PMC2768159

[102] Odegaard JI, Chawla A. Pleiotropic actions of insulin resistance and inflammation in metabolic homeostasis. Science. 2013;339(6116):172–7. 23307735 10.1126/science.1230721 PMC3725457

[103] Sun K, Kusminski CM, Scherer PE. Adipose tissue remodeling and obesity. J Clin Invest. 2011;121(6):2094–101. 21633177 10.1172/JCI45887 PMC3104761

[104] Takikawa A, Mahmood A, Nawaz A, Kado T, Okabe K, Yamamoto S, HIF-1alpha in Myeloid Cells Promotes Adipose Tissue Remodeling Toward Insulin Resistance. Diabetes. 2016;65(12):3649–59. 27625023 10.2337/db16-0012

[105] Lacasa D, Taleb S, Keophiphath M, Miranville A, Clement K. Macrophage-secreted factors impair human adipogenesis: involvement of proinflammatory state in preadipocytes. Endocrinology. 2007;148(2):868–77. 17082259 10.1210/en.2006-0687

[106] Qiu Y, Nguyen KD, Odegaard JI, Cui X, Tian X, Locksley RM, Eosinophils and type 2 cytokine signaling in macrophages orchestrate development of functional beige fat. Cell. 2014;157(6):1292–308. 24906148 10.1016/j.cell.2014.03.066 PMC4129510

[107] van den Berg SM, van Dam AD, Rensen PC, de Winther MP, Lutgens E. Immune Modulation of Brown(ing) Adipose Tissue in Obesity. Endocr Rev. 2017;38(1):46–68. 27849358 10.1210/er.2016-1066

[108] Fischer K, Ruiz HH, Jhun K, Finan B, Oberlin DJ, van der Heide V, Alternatively activated macrophages do not synthesize catecholamines or contribute to adipose tissue adaptive thermogenesis. Nat Med. 2017;23(5):623–30. 28414329 10.1038/nm.4316 PMC5420449

[109] Lee MW, Odegaard JI, Mukundan L, Qiu Y, Molofsky AB, Nussbaum JC, Activated type 2 innate lymphoid cells regulate beige fat biogenesis. Cell. 2015;160(1–2):74–87. 25543153 10.1016/j.cell.2014.12.011 PMC4297518

[110] Lizcano F, Vargas D, Gomez A, Torrado A. Human ADMC-Derived Adipocyte Thermogenic Capacity Is Regulated by IL-4 Receptor. Stem Cells Int. 2017;2017:2767916. 29158739 10.1155/2017/2767916 PMC5660824

[111] Nawaz A, Aminuddin A, Kado T, Takikawa A, Yamamoto S, Tsuneyama K, CD206(+) M2-like macrophages regulate systemic glucose metabolism by inhibiting proliferation of adipocyte progenitors. Nat Commun. 2017;8(1):286. 28819169 10.1038/s41467-017-00231-1 PMC5561263

[112] Ignotz RA, Massague J. Type beta transforming growth factor controls the adipogenic differentiation of 3T3 fibroblasts. Proc Natl Acad Sci U S A. 1985;82(24):8530–4. 3001708 10.1073/pnas.82.24.8530 PMC390950

[113] Yadav H, Rane SG. TGF-beta/Smad3 Signaling Regulates Brown Adipocyte Induction in White Adipose Tissue. Front Endocrinol (Lausanne). 2012;3:35. 22654861 10.3389/fendo.2012.00035 PMC3356034

[114] Zeve D, Seo J, Suh JM, Stenesen D, Tang W, Berglund ED, Wnt signaling activation in adipose progenitors promotes insulin-independent muscle glucose uptake. Cell Metab. 2012;15(4):492–504. 22482731 10.1016/j.cmet.2012.03.010 PMC3325026

[115] Longo KA, Wright WS, Kang S, Gerin I, Chiang SH, Lucas PC, Wnt10b inhibits development of white and brown adipose tissues. J Biol Chem. 2004;279(34):35503–9. 15190075 10.1074/jbc.M402937200

[116] Kakudo N, Shimotsuma A, Kusumoto K. Fibroblast growth factor-2 stimulates adipogenic differentiation of human adipose-derived stem cells. Biochem Biophys Res Commun. 2007;359(2):239–44. 17543283 10.1016/j.bbrc.2007.05.070

[117] Zaragosi LE, Ailhaud G, Dani C. Autocrine fibroblast growth factor 2 signaling is critical for self-renewal of human multipotent adipose-derived stem cells. Stem Cells. 2006;24(11):2412–9. 16840552 10.1634/stemcells.2006-0006

[118] Rider DA, Dombrowski C, Sawyer AA, Ng GH, Leong D, Hutmacher DW, Autocrine fibroblast growth factor 2 increases the multipotentiality of human adipose-derived mesenchymal stem cells. Stem Cells. 2008;26(6):1598–608. 18356575 10.1634/stemcells.2007-0480

[119] Ma Y, Kakudo N, Morimoto N, Lai F, Taketani S, Kusumoto K. Fibroblast growth factor-2 stimulates proliferation of human adipose-derived stem cells via Src activation. Stem Cell Res Ther. 2019;10(1):350. 31775870 10.1186/s13287-019-1462-z PMC6882332

[120] Oliva-Olivera W, Coin-Araguez L, Lhamyani S, Clemente-Postigo M, Torres JA, Bernal-Lopez MR, Adipogenic Impairment of Adipose Tissue-Derived Mesenchymal Stem Cells in Subjects With Metabolic Syndrome: Possible Protective Role of FGF2. J Clin Endocrinol Metab. 2017;102(2):478–87. 27967316 10.1210/jc.2016-2256

[121] Cheng Y, Lin KH, Young TH, Cheng NC. The influence of fibroblast growth factor 2 on the senescence of human adipose-derived mesenchymal stem cells during long-term culture. Stem Cells Transl Med. 2020;9(4):518–530. 31840944 10.1002/sctm.19-0234 PMC7103622

[122] Patel NG, Kumar S, Eggo MC. Essential role of fibroblast growth factor signaling in preadipoctye differentiation. J Clin Endocrinol Metab. 2005;90(2):1226–32. 15522930 10.1210/jc.2004-1309

[123] Ohta H, Itoh N. Roles of FGFs as Adipokines in Adipose Tissue Development, Remodeling, and Metabolism. Front Endocrinol (Lausanne). 2014;5:18. 24605108 10.3389/fendo.2014.00018 PMC3932445

[124] Konishi M, Asaki T, Koike N, Miwa H, Miyake A, Itoh N. Role of Fgf10 in cell proliferation in white adipose tissue. Mol Cell Endocrinol. 2006;249(1–2):71–7. 16513252 10.1016/j.mce.2006.01.010

[125] Liu C, Meng M, Xu B, Xu Y, Li G, Cao Y, Fibroblast Growth Factor 6 Promotes Adipocyte Progenitor Cell Proliferation for Adipose Tissue Homeostasis. Diabetes. 2023;72(4):467–82. 36607240 10.2337/db22-0585

[126] Huang H, Song TJ, Li X, Hu L, He Q, Liu M, BMP signaling pathway is required for commitment of C3H10T1/2 pluripotent stem cells to the adipocyte lineage. Proc Natl Acad Sci U S A. 2009;106(31):12670–5. 19620713 10.1073/pnas.0906266106 PMC2722335

[127] Tang QQ, Otto TC, Lane MD. Commitment of C3H10T1/2 pluripotent stem cells to the adipocyte lineage. Proc Natl Acad Sci U S A. 2004;101(26):9607–11. 15210946 10.1073/pnas.0403100101 PMC470722

[128] Modica S, Straub LG, Balaz M, Sun W, Varga L, Stefanicka P, Bmp4 Promotes a Brown to White-like Adipocyte Shift. Cell Rep. 2016;16(8):2243–58. 27524617 10.1016/j.celrep.2016.07.048

[129] Tseng YH, Kokkotou E, Schulz TJ, Huang TL, Winnay JN, Taniguchi CM, New role of bone morphogenetic protein 7 in brown adipogenesis and energy expenditure. Nature. 2008;454(7207):1000–4. 18719589 10.1038/nature07221 PMC2745972

[130] Sottile V, Seuwen K. Bone morphogenetic protein-2 stimulates adipogenic differentiation of mesenchymal precursor cells in synergy with BRL 49653 (rosiglitazone). FEBS Lett. 2000;475(3):201–4. 10869556 10.1016/s0014-5793(00)01655-0

[131] Hata K, Nishimura R, Ikeda F, Yamashita K, Matsubara T, Nokubi T, Differential roles of Smad1 and p38 kinase in regulation of peroxisome proliferator-activating receptor gamma during bone morphogenetic protein 2-induced adipogenesis. Mol Biol Cell. 2003;14(2):545–55. 12589053 10.1091/mbc.E02-06-0356 PMC149991

[132] Jin W, Takagi T, Kanesashi SN, Kurahashi T, Nomura T, Harada J, Schnurri-2 controls BMP-dependent adipogenesis via interaction with Smad proteins. Dev Cell. 2006;10(4):461–71. 16580992 10.1016/j.devcel.2006.02.016

[133] Denton NF, Eghleilib M, Al-Sharifi S, Todorcevic M, Neville MJ, Loh N, Bone morphogenetic protein 2 is a depot-specific regulator of human adipogenesis. Int J Obes (Lond). 2019;43(12):2458–68. 31324879 10.1038/s41366-019-0421-1 PMC6892741

[134] Shungin D, Winkler TW, Croteau-Chonka DC, Ferreira T, Locke AE, Magi R, New genetic loci link adipose and insulin biology to body fat distribution. Nature. 2015;518(7538):187–96. 25673412 10.1038/nature14132 PMC4338562

[135] Guiu-Jurado E, Unthan M, Bohler N, Kern M, Landgraf K, Dietrich A, Bone morphogenetic protein 2 (BMP2) may contribute to partition of energy storage into visceral and subcutaneous fat depots. Obesity (Silver Spring). 2016;24(10):2092–100. 27515773 10.1002/oby.21571

[136] Bowers RR, Kim JW, Otto TC, Lane MD. Stable stem cell commitment to the adipocyte lineage by inhibition of DNA methylation: role of the BMP-4 gene. Proc Natl Acad Sci U S A. 2006;103(35):13022–7. 16916928 10.1073/pnas.0605789103 PMC1559746

[137] Qian SW, Tang Y, Li X, Liu Y, Zhang YY, Huang HY, BMP4-mediated brown fat-like changes in white adipose tissue alter glucose and energy homeostasis. Proc Natl Acad Sci U S A. 2013;110(9):E798–807. 23388637 10.1073/pnas.1215236110 PMC3587258

[138] Gustafson B, Smith U. The WNT inhibitor Dickkopf 1 and bone morphogenetic protein 4 rescue adipogenesis in hypertrophic obesity in humans. Diabetes. 2012;61(5):1217–24. 22447857 10.2337/db11-1419 PMC3331742

[139] Gustafson B, Hammarstedt A, Hedjazifar S, Hoffmann JM, Svensson PA, Grimsby J, BMP4 and BMP Antagonists Regulate Human White and Beige Adipogenesis. Diabetes. 2015;64(5):1670–81. 25605802 10.2337/db14-1127

[140] Modica S, Wolfrum C. The dual role of BMP4 in adipogenesis and metabolism. Adipocyte. 2017;6(2):141–6. 28425843 10.1080/21623945.2017.1287637 PMC5477726

[141] Qian SW, Wu MY, Wang YN, Zhao YX, Zou Y, Pan JB, BMP4 facilitates beige fat biogenesis via regulating adipose tissue macrophages. J Mol Cell Biol. 2019;11(1):14–25. 29462349 10.1093/jmcb/mjy011 PMC6512770

[142] Townsend KL, Suzuki R, Huang TL, Jing E, Schulz TJ, Lee K, Bone morphogenetic protein 7 (BMP7) reverses obesity and regulates appetite through a central mTOR pathway. FASEB journal : official publication of the Federation of American Societies for Experimental Biology. 2012;26(5):2187–96. 22331196 10.1096/fj.11-199067 PMC3336788

[143] Schulz TJ, Huang P, Huang TL, Xue R, McDougall LE, Townsend KL, Brown-fat paucity due to impaired BMP signalling induces compensatory browning of white fat. Nature. 2013;495(7441):379–83. 23485971 10.1038/nature11943 PMC3623555

[144] Schulz TJ, Huang TL, Tran TT, Zhang H, Townsend KL, Shadrach JL, Identification of inducible brown adipocyte progenitors residing in skeletal muscle and white fat. Proceedings of the National Academy of Sciences of the United States of America. 2011;108(1):143–8. 21173238 10.1073/pnas.1010929108 PMC3017184

[145] Elsen M, Raschke S, Tennagels N, Schwahn U, Jelenik T, Roden M, BMP4 and BMP7 induce the white-to-brown transition of primary human adipose stem cells. Am J Physiol Cell Physiol. 2014;306(5):C431–40. 24284793 10.1152/ajpcell.00290.2013

[146] Nishio M, Yoneshiro T, Nakahara M, Suzuki S, Saeki K, Hasegawa M, Production of functional classical brown adipocytes from human pluripotent stem cells using specific hemopoietin cocktail without gene transfer. Cell metabolism. 2012;16(3):394–406. 22958922 10.1016/j.cmet.2012.08.001

[147] Blazquez-Medela AM, Jumabay M, Bostrom KI. Beyond the bone: Bone morphogenetic protein signaling in adipose tissue. Obes Rev. 2019;20(5):648–58. 30609449 10.1111/obr.12822 PMC6447448

[148] Rajakumari S, Wu J, Ishibashi J, Lim HW, Giang AH, Won KJ, EBF2 determines and maintains brown adipocyte identity. Cell Metab. 2013;17(4):562–74. 23499423 10.1016/j.cmet.2013.01.015 PMC3622114

[149] Wang W, Kissig M, Rajakumari S, Huang L, Lim HW, Won KJ, Ebf2 is a selective marker of brown and beige adipogenic precursor cells. Proc Natl Acad Sci U S A. 2014;111(40):14466–71. 25197048 10.1073/pnas.1412685111 PMC4209986

[150] Stine RR, Shapira SN, Lim HW, Ishibashi J, Harms M, Won KJ, EBF2 promotes the recruitment of beige adipocytes in white adipose tissue. Mol Metab. 2016;5(1):57–65. 26844207 10.1016/j.molmet.2015.11.001 PMC4703852

[151] Shao M, Ishibashi J, Kusminski CM, Wang QA, Hepler C, Vishvanath L, Zfp423 Maintains White Adipocyte Identity through Suppression of the Beige Cell Thermogenic Gene Program. Cell Metab. 2016;23(6):1167–84. 27238639 10.1016/j.cmet.2016.04.023 PMC5091077

[152] Chen C, Grzegorzewski KJ, Barash S, Zhao Q, Schneider H, Wang Q, An integrated functional genomics screening program reveals a role for BMP-9 in glucose homeostasis. Nat Biotechnol. 2003;21(3):294–301. 12598908 10.1038/nbt795

[153] Kuo MM, Kim S, Tseng CY, Jeon YH, Choe S, Lee DK. BMP-9 as a potent brown adipogenic inducer with anti-obesity capacity. Biomaterials. 2014;35(10):3172–9. 24439409 10.1016/j.biomaterials.2013.12.063

[154] Um JH, Park SY, Hur JH, Lee HY, Jeong KH, Cho Y, Bone morphogenic protein 9 is a novel thermogenic hepatokine secreted in response to cold exposure. Metabolism. 2022;129:155139. 35063533 10.1016/j.metabol.2022.155139

[155] Bai Y, Shang Q, Zhao H, Pan Z, Guo C, Zhang L, Pdcd4 restrains the self-renewal and white-to-beige transdifferentiation of adipose-derived stem cells. Cell Death Dis. 2016;7:e2169. 27031966 10.1038/cddis.2016.75 PMC4823969

[156] Boyer LA, Lee TI, Cole MF, Johnstone SE, Levine SS, Zucker JP, Core transcriptional regulatory circuitry in human embryonic stem cells. Cell. 2005;122(6):947–56. 16153702 10.1016/j.cell.2005.08.020 PMC3006442

[157] Wang J, Rao S, Chu J, Shen X, Levasseur DN, Theunissen TW, A protein interaction network for pluripotency of embryonic stem cells. Nature. 2006;444(7117):364–8. 17093407 10.1038/nature05284

[158] Sachs PC, Francis MP, Zhao M, Brumelle J, Rao RR, Elmore LW, Defining essential stem cell characteristics in adipose-derived stromal cells extracted from distinct anatomical sites. Cell Tissue Res. 2012;349(2):505–15. 22628159 10.1007/s00441-012-1423-7 PMC3746068

[159] Potdar P, Sutar J. Establishment and molecular characterization of mesenchymal stem cell lines derived from human visceral & subcutaneous adipose tissues. J Stem Cells Regen Med. 2010;6(1):26–35. 10.46582/jsrm.0601005 PMC3908252 24693057

[160] Taha MF, Javeri A, Rohban S, Mowla SJ. Upregulation of pluripotency markers in adipose tissue-derived stem cells by miR-302 and leukemia inhibitory factor. Biomed Res Int. 2014;2014:941486. 25147827 10.1155/2014/941486 PMC4132412

[161] Higuchi A, Wang CT, Ling QD, Lee HH, Kumar SS, Chang Y, A hybrid-membrane migration method to isolate high-purity adipose-derived stem cells from fat tissues. Sci Rep. 2015;5:10217. 25970301 10.1038/srep10217 PMC4429558

[162] Chen PM, Lin CH, Li NT, Wu YM, Lin MT, Hung SC, c-Maf regulates pluripotency genes, proliferation/self-renewal, and lineage commitment in ROS-mediated senescence of human mesenchymal stem cells. Oncotarget. 2015;6(34):35404–18. 26496036 10.18632/oncotarget.6178 PMC4742114

[163] Kim JH, Jee MK, Lee SY, Han TH, Kim BS, Kang KS, Regulation of adipose tissue stromal cells behaviors by endogenic Oct4 expression control. PLoS One. 2009;4(9):e7166. 19777066 10.1371/journal.pone.0007166 PMC2747014

[164] Pitrone M, Pizzolanti G, Tomasello L, Coppola A, Morini L, Pantuso G, NANOG Plays a Hierarchical Role in the Transcription Network Regulating the Pluripotency and Plasticity of Adipose Tissue-Derived Stem Cells. Int J Mol Sci. 2017;18(6). 10.3390/ijms18061107 PMC5485931 28545230

[165] Tsai CC, Su PF, Huang YF, Yew TL, Hung SC. Oct4 and Nanog directly regulate Dnmt1 to maintain self-renewal and undifferentiated state in mesenchymal stem cells. Mol Cell. 2012;47(2):169–82. 22795133 10.1016/j.molcel.2012.06.020

[166] Pitrone M, Pizzolanti G, Coppola A, Tomasello L, Martorana S, Pantuso G, Knockdown of NANOG Reduces Cell Proliferation and Induces G0/G1 Cell Cycle Arrest in Human Adipose Stem Cells. Int J Mol Sci. 2019;20(10):2580. 31130693 10.3390/ijms20102580 PMC6566573

[167] Wang Y, Kim KA, Kim JH, Sul HS. Pref-1, a preadipocyte secreted factor that inhibits adipogenesis. J Nutr. 2006;136(12):2953–6. 17116701 10.1093/jn/136.12.2953

[168] Mitterberger MC, Lechner S, Mattesich M, Kaiser A, Probst D, Wenger N, DLK1(PREF1) is a negative regulator of adipogenesis in CD105(+)/CD90(+)/CD34(+)/CD31(−)/FABP4(−) adipose-derived stromal cells from subcutaneous abdominal fat pats of adult women. Stem Cell Res. 2012;9(1):35–48. 22640926 10.1016/j.scr.2012.04.001

[169] Wang Q, Dong Z, Liu X, Song X, Song Q, Shang Q, Programmed cell death-4 deficiency prevents diet-induced obesity, adipose tissue inflammation, and insulin resistance. Diabetes. 2013;62(12):4132–43. 23990362 10.2337/db13-0097 PMC3837052

[170] Dao LT, Park EY, Hwang OK, Cha JY, Jun HS. Differentiation potential and profile of nuclear receptor expression during expanded culture of human adipose tissue-derived stem cells reveals PPARgamma as an important regulator of Oct4 expression. Stem Cells Dev. 2014;23(1):24–33. 10.1089/scd.2013.0137 23998797

[171] Kapetanou M, Chondrogianni N, Petrakis S, Koliakos G, Gonos ES. Proteasome activation enhances stemness and lifespan of human mesenchymal stem cells. Free Radic Biol Med. 2017;103:226–35. 28034832 10.1016/j.freeradbiomed.2016.12.035

[172] Wang QA, Tao C, Gupta RK, Scherer PE. Tracking adipogenesis during white adipose tissue development, expansion and regeneration. Nat Med. 2013;19(10):1338–44. 23995282 10.1038/nm.3324 PMC4075943

[173] Arner P, Andersson DP, Thorne A, Wiren M, Hoffstedt J, Naslund E, Variations in the size of the major omentum are primarily determined by fat cell number. J Clin Endocrinol Metab. 2013;98(5):E897–901. 23543656 10.1210/jc.2012-4106

[174] Onate B, Vilahur G, Ferrer-Lorente R, Ybarra J, Diez-Caballero A, Ballesta-Lopez C, The subcutaneous adipose tissue reservoir of functionally active stem cells is reduced in obese patients. FASEB J. 2012;26(10):4327–36. 22772162 10.1096/fj.12-207217

[175] Shang Q, Bai Y, Wang G, Song Q, Guo C, Zhang L, Delivery of Adipose-Derived Stem Cells Attenuates Adipose Tissue Inflammation and Insulin Resistance in Obese Mice Through Remodeling Macrophage Phenotypes. Stem Cells Dev. 2015;24(17):2052–64. 25923535 10.1089/scd.2014.0557

[176] Frazier TP, Gimble JM, Devay JW, Tucker HA, Chiu ES, Rowan BG. Body mass index affects proliferation and osteogenic differentiation of human subcutaneous adipose tissue-derived stem cells. BMC Cell Biol. 2013;14:34. 23924189 10.1186/1471-2121-14-34 PMC3750383

[177] Roldan M, Macias-Gonzalez M, Garcia R, Tinahones FJ, Martin M. Obesity short-circuits stemness gene network in human adipose multipotent stem cells. FASEB J. 2011;25(12):4111–26. 21846837 10.1096/fj.10-171439

[178] Perez LM, Bernal A, San Martin N, Galvez BG. Obese-derived ASCs show impaired migration and angiogenesis properties. Arch Physiol Biochem. 2013;119(5):195–201. 23672297 10.3109/13813455.2013.784339 PMC3836426

[179] Zheng G, Qiu G, Ge M, He J, Huang L, Chen P, Human adipose-derived mesenchymal stem cells alleviate obliterative bronchiolitis in a murine model via IDO. Respir Res. 2017;18(1):119. 28619045 10.1186/s12931-017-0599-5 PMC5472885

[180] Spaggiari GM, Moretta L. Cellular and molecular interactions of mesenchymal stem cells in innate immunity. Immunol Cell Biol. 2013;91(1):27–31. 23146943 10.1038/icb.2012.62

[181] Serena C, Keiran N, Ceperuelo-Mallafre V, Ejarque M, Fradera R, Roche K, Obesity and Type 2 Diabetes Alters the Immune Properties of Human Adipose Derived Stem Cells. Stem Cells. 2016;34(10):2559–73. 27352919 10.1002/stem.2429

[182] Silva KR, Liechocki S, Carneiro JR, Claudio-da-Silva C, Maya-Monteiro CM, Borojevic R, Stromal-vascular fraction content and adipose stem cell behavior are altered in morbid obese and post bariatric surgery ex-obese women. Stem Cell Res Ther. 2015;6:72. 25884374 10.1186/s13287-015-0029-x PMC4435525

[183] Perez LM, de Lucas B, Lunyak VV, Galvez BG. Adipose stem cells from obese patients show specific differences in the metabolic regulators vitamin D and Gas5. Mol Genet Metab Rep. 2017;12:51–6. 28580301 10.1016/j.ymgmr.2017.05.008 PMC5447652

[184] Lee MJ, Kim J, Kim MY, Bae YS, Ryu SH, Lee TG, Proteomic analysis of tumor necrosis factor-alpha-induced secretome of human adipose tissue-derived mesenchymal stem cells. J Proteome Res. 2010;9(4):1754–62. 20184379 10.1021/pr900898n

[185] Kilroy GE, Foster SJ, Wu X, Ruiz J, Sherwood S, Heifetz A, Cytokine profile of human adipose-derived stem cells: expression of angiogenic, hematopoietic, and pro-inflammatory factors. J Cell Physiol. 2007;212(3):702–9. 17477371 10.1002/jcp.21068

[186] Patel RS, Carter G, El Bassit G, Patel AA, Cooper DR, Murr M, Adipose-derived stem cells from lean and obese humans show depot specific differences in their stem cell markers, exosome contents and senescence: role of protein kinase C delta (PKCdelta) in adipose stem cell niche. Stem Cell Investig. 2016;3:2. 10.3978/j.issn.2306-9759.2016.01.02 PMC4923648 27358894

[187] Dentelli P, Barale C, Togliatto G, Trombetta A, Olgasi C, Gili M, A diabetic milieu promotes OCT4 and NANOG production in human visceral-derived adipose stem cells. Diabetologia. 2013;56(1):173–84. 23064289 10.1007/s00125-012-2734-7

[188] Perez LM, Bernal A, de Lucas B, San Martin N, Mastrangelo A, Garcia A, Altered metabolic and stemness capacity of adipose tissue-derived stem cells from obese mouse and human. PLoS One. 2015;10(4):e0123397. 25875023 10.1371/journal.pone.0123397 PMC4395137

[189] Gao Z, Daquinag AC, Fussell C, Zhao Z, Dai Y, Rivera A, Age-associated telomere attrition in adipocyte progenitors predisposes to metabolic disease. Nat Metab. 2020;2(12):1482–97. 33324010 10.1038/s42255-020-00320-4

[190] Ritter A, Friemel A, Kreis NN, Hoock SC, Roth S, Kielland-Kaisen U, Primary Cilia Are Dysfunctional in Obese Adipose-Derived Mesenchymal Stem Cells. Stem Cell Reports. 2018;10(2):583–99. 29396182 10.1016/j.stemcr.2017.12.022 PMC5830986

[191] Ritter A, Kreis NN, Roth S, Friemel A, Jennewein L, Eichbaum C, Restoration of primary cilia in obese adipose-derived mesenchymal stem cells by inhibiting Aurora A or extracellular signal-regulated kinase. Stem Cell Res Ther. 2019;10(1):255. 31412932 10.1186/s13287-019-1373-z PMC6694567

[192] Badimon L, Cubedo J. Adipose tissue depots and inflammation: effects on plasticity and resident mesenchymal stem cell function. Cardiovasc Res. 2017;113(9):1064–73. 28498891 10.1093/cvr/cvx096

[193] Mika A, Macaluso F, Barone R, Di Felice V, Sledzinski T. Effect of Exercise on Fatty Acid Metabolism and Adipokine Secretion in Adipose Tissue. Front Physiol. 2019;10:26. 30745881 10.3389/fphys.2019.00026 PMC6360148

[194] Loustau T, Coudiere E, Karkeni E, Landrier JF, Jover B, Riva C. Murine double minute-2 mediates exercise-induced angiogenesis in adipose tissue of diet-induced obese mice. Microvasc Res. 2020;130:104003. 32199946 10.1016/j.mvr.2020.104003

[195] Woo J, Kang S. Diet change and exercise enhance protein expression of CREB, CRTC 2 and lipolitic enzymes in adipocytes of obese mice. Lipids Health Dis. 2016;15(1):147. 27596982 10.1186/s12944-016-0316-2 PMC5011960

[196] Giles ED, Steig AJ, Jackman MR, Higgins JA, Johnson GC, Lindstrom RC, Exercise Decreases Lipogenic Gene Expression in Adipose Tissue and Alters Adipocyte Cellularity during Weight Regain After Weight Loss. Front Physiol. 2016;7:32. 26903882 10.3389/fphys.2016.00032 PMC4748045

[197] Buffolo M, Pires KM, Ferhat M, Ilkun O, Makaju A, Achenbach A, Identification of a Paracrine Signaling Mechanism Linking CD34(high) Progenitors to the Regulation of Visceral Fat Expansion and Remodeling. Cell Rep. 2019;29(2):270–82 e5. 31597091 10.1016/j.celrep.2019.08.092 PMC10950319

[198] Hepler C, Shan B, Zhang Q, Henry GH, Shao M, Vishvanath L, Identification of functionally distinct fibro-inflammatory and adipogenic stromal subpopulations in visceral adipose tissue of adult mice. Elife. 2018;7. 10.7554/eLife.39636 PMC6167054 30265241

[199] Marcelin G, Ferreira A, Liu Y, Atlan M, Aron-Wisnewsky J, Pelloux V, A PDGFRalpha-Mediated Switch toward CD9(high) Adipocyte Progenitors Controls Obesity-Induced Adipose Tissue Fibrosis. Cell Metab. 2017;25(3):673–85. 28215843 10.1016/j.cmet.2017.01.010

[200] Raajendiran A, Ooi G, Bayliss J, O’Brien PE, Schittenhelm RB, Clark AK, Identification of Metabolically Distinct Adipocyte Progenitor Cells in Human Adipose Tissues. Cell Rep. 2019;27(5):1528–40 e7. 31042478 10.1016/j.celrep.2019.04.010

[201] Ludzki AC, Krueger EM, Baldwin TC, Schleh MW, Porsche CE, Ryan BJ, Acute Aerobic Exercise Remodels the Adipose Tissue Progenitor Cell Phenotype in Obese Adults. Front Physiol. 2020;11:903. 32848853 10.3389/fphys.2020.00903 PMC7399179

[202] Kolahdouzi S, Talebi-Garakani E, Hamidian G, Safarzade A. Exercise training prevents high-fat diet-induced adipose tissue remodeling by promoting capillary density and macrophage polarization. Life Sci. 2019;220:32–43. 30690082 10.1016/j.lfs.2019.01.037

[203] Honkala SM, Motiani P, Kivela R, Hemanthakumar KA, Tolvanen E, Motiani KK, Exercise training improves adipose tissue metabolism and vasculature regardless of baseline glucose tolerance and sex. BMJ Open Diabetes Res Care. 2020;8(1):e000830. 10.1136/bmjdrc-2019-000830 PMC7437884 32816872

[204] Geng L, Liao B, Jin L, Huang Z, Triggle CR, Ding H, Exercise Alleviates Obesity-Induced Metabolic Dysfunction via Enhancing FGF21 Sensitivity in Adipose Tissues. Cell Rep. 2019;26(10):2738–52 e4. 30840894 10.1016/j.celrep.2019.02.014

[205] Kawanishi N, Niihara H, Mizokami T, Yano H, Suzuki K. Exercise training attenuates adipose tissue fibrosis in diet-induced obese mice. Biochem Biophys Res Commun. 2013;440(4):774–9. 24120495 10.1016/j.bbrc.2013.10.004

[206] Weindruch R, Sohal RS. Seminars in medicine of the Beth Israel Deaconess Medical Center. Caloric intake and aging. N Engl J Med. 1997;337(14):986–94. 9309105 10.1056/NEJM199710023371407 PMC2851235

[207] Larson-Meyer DE, Heilbronn LK, Redman LM, Newcomer BR, Frisard MI, Anton S, Effect of calorie restriction with or without exercise on insulin sensitivity, beta-cell function, fat cell size, and ectopic lipid in overweight subjects. Diabetes Care. 2006;29(6):1337–44. 16732018 10.2337/dc05-2565 PMC2677812

[208] Fabbiano S, Suarez-Zamorano N, Rigo D, Veyrat-Durebex C, Stevanovic Dokic A, Colin DJ, Caloric Restriction Leads to Browning of White Adipose Tissue through Type 2 Immune Signaling. Cell Metab. 2016;24(3):434–46. 27568549 10.1016/j.cmet.2016.07.023

[209] Anderson RM, Barger JL, Edwards MG, Braun KH, O’Connor CE, Prolla TA, Dynamic regulation of PGC-1alpha localization and turnover implicates mitochondrial adaptation in calorie restriction and the stress response. Aging Cell. 2008;7(1):101–11. 18031569 10.1111/j.1474-9726.2007.00357.x PMC2253697

[210] Fisher FM, Kleiner S, Douris N, Fox EC, Mepani RJ, Verdeguer F, FGF21 regulates PGC-1alpha and browning of white adipose tissues in adaptive thermogenesis. Genes Dev. 2012;26(3):271–81. 22302939 10.1101/gad.177857.111 PMC3278894

[211] Fujii N, Uta S, Kobayashi M, Sato T, Okita N, Higami Y. Impact of aging and caloric restriction on fibroblast growth factor 21 signaling in rat white adipose tissue. Exp Gerontol. 2019;118:55–64. 30620889 10.1016/j.exger.2019.01.001

[212] Barquissau V, Leger B, Beuzelin D, Martins F, Amri EZ, Pisani DF, Caloric Restriction and Diet-Induced Weight Loss Do Not Induce Browning of Human Subcutaneous White Adipose Tissue in Women and Men with Obesity. Cell Rep. 2018;22(4):1079–89. 29386128 10.1016/j.celrep.2017.12.102

[213] Dai R, Wang Z, Samanipour R, Koo KI, Kim K. Adipose-Derived Stem Cells for Tissue Engineering and Regenerative Medicine Applications. Stem Cells Int. 2016;2016:6737345. 27057174 10.1155/2016/6737345 PMC4761677

[214] Turtzo LC, Marx R, Lane MD. Cross-talk between sympathetic neurons and adipocytes in coculture. Proc Natl Acad Sci U S A. 2001;98(22):12385–90. 11606782 10.1073/pnas.231478898 PMC60063

[215] Wankhade UD, Shen M, Kolhe R, Fulzele S. Advances in Adipose-Derived Stem Cells Isolation, Characterization, and Application in Regenerative Tissue Engineering. Stem Cells Int. 2016;2016:3206807. 26981130 10.1155/2016/3206807 PMC4766348

[216] Brown AC. Insights into the adipose stem cell niche in health and disease. In: Kokai L, Marra K, Rubin JP, editors. Scientific Principles of Adipose Stem Cells. Academic Press; 2022. p. 57–80. Copyright Elsevier.

